# Import, use, and emissions of PCBs in Switzerland from 1930 to 2100

**DOI:** 10.1371/journal.pone.0183768

**Published:** 2017-10-05

**Authors:** Juliane Glüge, Christine Steinlin, Simone Schalles, Lukas Wegmann, Josef Tremp, Knut Breivik, Konrad Hungerbühler, Christian Bogdal

**Affiliations:** 1 Institute for Chemical and Bioengineering, Swiss Federal Institute of Technology, ETH Zurich, Zurich, Switzerland; 2 Office for Environmental Protection and Energy of the Canton Basel-Country, Liestal, Switzerland; 3 Industrial Chemicals Section, Federal Office for the Environment, Bern, Switzerland; 4 NILU - Norwegian Institute for Air Research, Kjeller, Norway; 5 University of Oslo, Department of Chemistry, Oslo, Norway; University of Iowa, UNITED STATES

## Abstract

Polychlorinated biphenyls (PCBs) are persistent organic compounds that are ubiquitously found in the environment. Their use and manufacture were restricted or banned in many countries in the 1970–1980s, however, they still persist in the antroposphere, the environment and in biota worldwide today. Conventions like the Convention on Long-range Transboundary Air Pollution encourage or bind the member parties to annually submit emission inventories of regulated air pollutants. Unfortunately, several member states have not yet reported PCB emissions. The identification and quantification of stocks and emissions sources is, however, an important precondition to handle and remove the remaining reservoirs of PCBs and, thus, to be able to reduce emissions and subsequently environmental exposure. Here, we estimate past, present, and future emissions of PCBs to air in Switzerland and provide emission factors for all relevant emission categories. Switzerland hereby represents a typical developed industrial country, and most of the assumptions and parameters presented here can be used to calculate PCB emission also for other countries. PCB emissions to air are calculated using a dynamic mass flow and emissions model for Switzerland, which is run for the years 1930–2100. The results point out the importance of the use of PCBs in open applications, which have largely been previously overlooked. Additionally, we show that PCBs will persist in applications during the coming decades with ongoing emissions. Especially the use of PCBs in open applications will cause Swiss emissions to remain above 100 kg PCB per year, even after the year 2030. Our developed model is available in Excel/VBA and can be downloaded with this article.

## Introduction

Polychlorinated biphenyls (PCBs) are anthropogenic compounds that are highly resistant to environmental degradation, accumulate in biota and humans, and have negative health effects. PCBs undergo long-range atmospheric transport, resulting in their global distribution in the environment [1–3]. After PCB use and manufacturing were banned in the 1980s in many countries, levels started to decline slowly in biota and in the abiotic environment around the world. However, several studies show a slowdown of the decreasing levels in recent years [4, 5]. PCBs persist, for example, at excessively high concentrations in some cetaceans, including killer whales and bottlenose dolphins in the northeast Atlantic and in many cetacean species in the Mediterranean Sea [6]. The measured high PCB concentrations in the European cetaceans from 1990 to 2012 were widely associated with long-term population declines and low or zero rates of reproduction, consistent with severe PCB-induced population-level effects [4].

The 1979 UNECE Convention on Long-range Transboundary Air Pollution (CLRTAP), also referred to as the Geneva Convention, aims at reducing the damage to the environment and human health caused by transboundary air pollution [7]. The 1998 Aarhus Protocol on Persistent Organic Pollutants to the CLRTAP binds the parties to annually submit emission inventories for substances listed in annex III, and encourages the parties to submit emission inventories for substances listed in annex I and II of the protocol, including PCBs [8, 9]. While the annual submission of emissions is very comprehensive for most of the pollutants targeted by the convention, the submission shows severe gaps for PCBs (see Section K in S1 File). One reason for this might be that the EMEP/EEA air pollutant emission inventory guidebook of the European Environment Agency is ‘incomplete’ in terms of source coverage for PCBs. The guidebook assumes, for example, that the majority of PCB emissions arises from leaks of PCB-containing large electrical transformers and capacitors and from the fragmentation of small capacitors [10]. PCB emissions from the use of open applications (anti-corrosive paints, joint sealants) are not considered in the guidebook. Additionally, the emission factors for leaks from transformers and capacitors and for fragmentising operations (recycling of ferrous scrap) are reported on a per capita basis and do not consider differences between the countries. Reported PCB emissions to air to the UNECE Secretariat of the CLRTAP based on the emission factors from the guidebook might therefore be highly uncertain and most probably underestimate the actual emissions. The reporting of PCB emissions under the CLRTAP is currently still voluntary, but the amended version of the Aarhus protocol, which is currently under ratification, will require the development and maintaining of emission inventories for more substances, including PCBs [11]. The identification and quantification of emissions sources of PCBs is therefore not only an important precondition to handle and remove the remaining reservoirs of PCBs, but will also be mandatory for the parties of the CLRTAP in the future. The objective of this study is to estimate past, present, and future emissions of PCBs to air in Switzerland and to provide emission factors for all relevant emission categories. PCBs can be emitted during their intentional use or by unintentional formation, for example, from the combustion of coal, oil, biogas, wood, or straw. Our study focuses on emissions from intentionally used PCBs because those are expected by far to exceed PCB emissions by unintentional formation. The Swiss Federal Office for the Environment (FOEN) estimated Swiss emissions to air from unintentional formation of wood and coal combustion for the sum of all PCB congeners between 1980 and 2013 to be in the range of 1.8 to 15.1 kg PCB per year [12]. This is less than 1% of the emissions we calculated in this study for the intentionally used PCBs in this time period. Similarly, unintentional emissions in China contributed less than 3% to the total historical Chinese PCB emissions [13]. However, it is important to note that the assumption that PCBs are mainly emitted from intentional uses refers to the sum of all PCB congeners. In contrast, single individual congeners (for example PCB-11) might be mainly emitted by unintentional formation [14].

Emissions to air in this study are calculated using a mass balance model for Switzerland, which is run for the years 1930–2100. PCB emissions were considered from the usage of transformers, large capacitors, small capacitors, anti-corrosive paints, and joint sealants as well as from accidental release, treatment, and disposal of PCBs. A similar mass balance model has previously been developed by Breivik et al. [15, 16], where the authors defined a default scenario using parameters from the literature, a high scenario where they increased emission parameters by factors of five to ten, and a low scenario where they decreased emission parameters. This model was originally developed to try to understand the ‘big picture’ of the historical global releases of PCBs (for example Arctic pollution by PCBs), however it is highly uncertain for a specific country and year. The high uncertainties for a specific country and year originate from a lack of detailed national information to reliably parametrise the stocks and emissions within individual countries. In our study, we quantify a detailed emission inventory for PCBs in Switzerland using country specific information where possible. Switzerland hereby represents a developed industrial country and most of the assumptions and parameters (for example emission factors) can be used for other countries. Using our study, other countries should now be able to set up a PCB emissions inventory and report PCB emissions. This might be especially important for countries that have not yet developed an emission inventory for PCBs, but will need to report PCB emissions to the UNECE secretary of the CLRTAP in the future. Our developed model is available in Excel/VBA and can be downloaded with this article (S2 File). The implemented model in MATLAB is available on request.

## PCBs

### PCB congeners

There are 209 individual PCB congeners with one to ten chlorine atoms attached to two benzene rings. These congeners have unique physical-chemical properties which define their environmental behaviour. In the model presented in this study, each of these 209 PCB congeners is tracked individually through the system, according to its properties. The presented emissions are the sum of the emissions of all 209 congeners.

The technical PCB products, formerly used in the different applications, contained congener mixtures with varying degrees of chlorination. This means that some products contained more of the higher-chlorinated congeners, while others contained more of the lower-chlorinated congeners. The six PCB congeners -28, -52, -101, -138, -153, and -180 were present in large fractions in these mixtures and are, therefore, commonly referred to as indicator PCB congeners (iPCBs). Their physical-chemical properties are well known, they are commonly measured in various samples, and they represent a wide range of properties. For the non-iPCB congeners without available data such as emission factors, data was derived from the iPCB, by interpolating the data using the chlorination degree of the congeners. The so-called dioxin-like PCBs (dl-PCBs) represent another commonly discussed group of 12 PCB congeners (PCB congeners -77, -81, -105, -114, -118, -123, -126, -156, -157, -167, -169, and -189), which are particularly important from a toxicological point of view, as they exhibit toxic effects similar to the polychlorinated dibenzo-*p*-dioxins. The toxicity of the dl-PCBs is expressed in toxic equivalency factors (TEFs), which express their toxicity in comparison to 2,3,7,8-tetrachlorodibenzo-*p*-dioxin (2,3,7,8-TCDD) [17, 18] (Section A in S1 File). The total toxic equivalent (TEQ) expresses then the total 2,3,7,8-TCDD-like activity of the mixture. The TEQ is calculated as:

$$\begin{array}{c} \text{TEQ}=\sum_{1}^{\text{dl-PCBs}}m_{\text{PCB}}\cdot {\text{TEF}}_{\text{PCB}} \end{array}$$ (1)

where *m* is the amount of the specific PCB congener in the mixture. For the mass budget of PCBs in technical applications, the dioxin-like PCBs are, however, of minor importance.

### Mixture for each use category

PCBs were typically used as mixtures of compounds where one mixture contained up to 130 individual congeners [19, 20]. As we wanted to track each PCB congener individually through the system, we needed to know which technical mixtures (with which PCB congeners) were used in which applications (use categories). PCB congeners which were not used in any of the mixtures assigned to a use category were not tracked through the model.

PCBs were used in Switzerland mainly in transformer askarels, in small and large capacitors, in joint sealants, and in anti-corrosive paints. Although, information on which technical mixtures were used in which applications, specifically in Switzerland, are missing. However, we assumed that the degree of chlorination was the most important property for the choice of a mixture in an application and that mixtures that were used in other countries for the same applications had similar portions of lower- and higher-chlorinated PCBs compared to mixtures used in Switzerland. This was also assumed by Breivik et al. [21] and is partly proven by Takasuga et al. [22]. Since most data are available for the Aroclors, these mixtures were used as ‘model mixtures’ across producers because of the similarities noted above.

The most common transformer askarels used in the US were Aroclor 1260 and Aroclor 1254 [23], so we therefore assumed the amount in use in transformers to be 50% Aroclor 1260 and 50% Aroclor 1254. Also in joint sealants, Aroclor 1254 and other mixtures with a high chlorination degree were used [23]. We assumed the amount in use in joint sealants to be 100% Aroclor 1254, as Erickson and Kaley [23] did not name the other technical mixtures. In capacitors, the main Aroclor formulation employed has been Aroclor 1016, although Aroclor 1242, 1254 and 1221 have also been used [24]. Harrad et al. [24] estimated in the absence of data concerning the relative distribution of individual congeners in Aroclor 1221, the relative distribution of Aroclors 1016, 1242 and 1254 in PCB-filled capacitors as being 8:1:1. We used the same ratio for the amount in use in capacitors in our study. For paints in general, various PCB mixtures were used [23], but mixtures with a high chlorination degree were mainly used for chlorinated rubber (for example Clophen A60) [25]. The company Bayer (which produced Clophen) stated that Clophen A60 was mainly used for open applications [25]. Based on this information, we assumed the amount in use in anti-corrosive paints to be 100% Clophen A60. The PCB congener compositions of the technical mixtures were taken from Frame et al. [19] and Taniyasu et al. [20]. Table 1 shows the fractions of the different congener groups in the mixtures used for the specific uses. We grouped the congeners here in congener groups according to their number of chlorine atoms to facilitate an overview.

**Table 1:** Fractions of the different congener groups in the mixtures used for the specific use categories [%].

PCB congeners	properties from	transformers	large capacitors	small capacitors	joint sealants	anti-corrosive paints
1-3 Cl	PCB-28	0.62	64.5	64.5	0.07	0.96
4 Cl	PCB-52	8.86	26.2	26.2	0.38	17.3
5 Cl	PCB-101	32.0	6.64	6.64	9.13	55.5
6 Cl	PCB-138	16.9	1.23	1.23	29.5	12.2
6 Cl	PCB-153	16.9	1.23	1.23	29.5	12.2
7 Cl	PCB-180	24.7	0.21	0.21	31.5	2.02

## Model setup

### Overview

**Fig 1:**
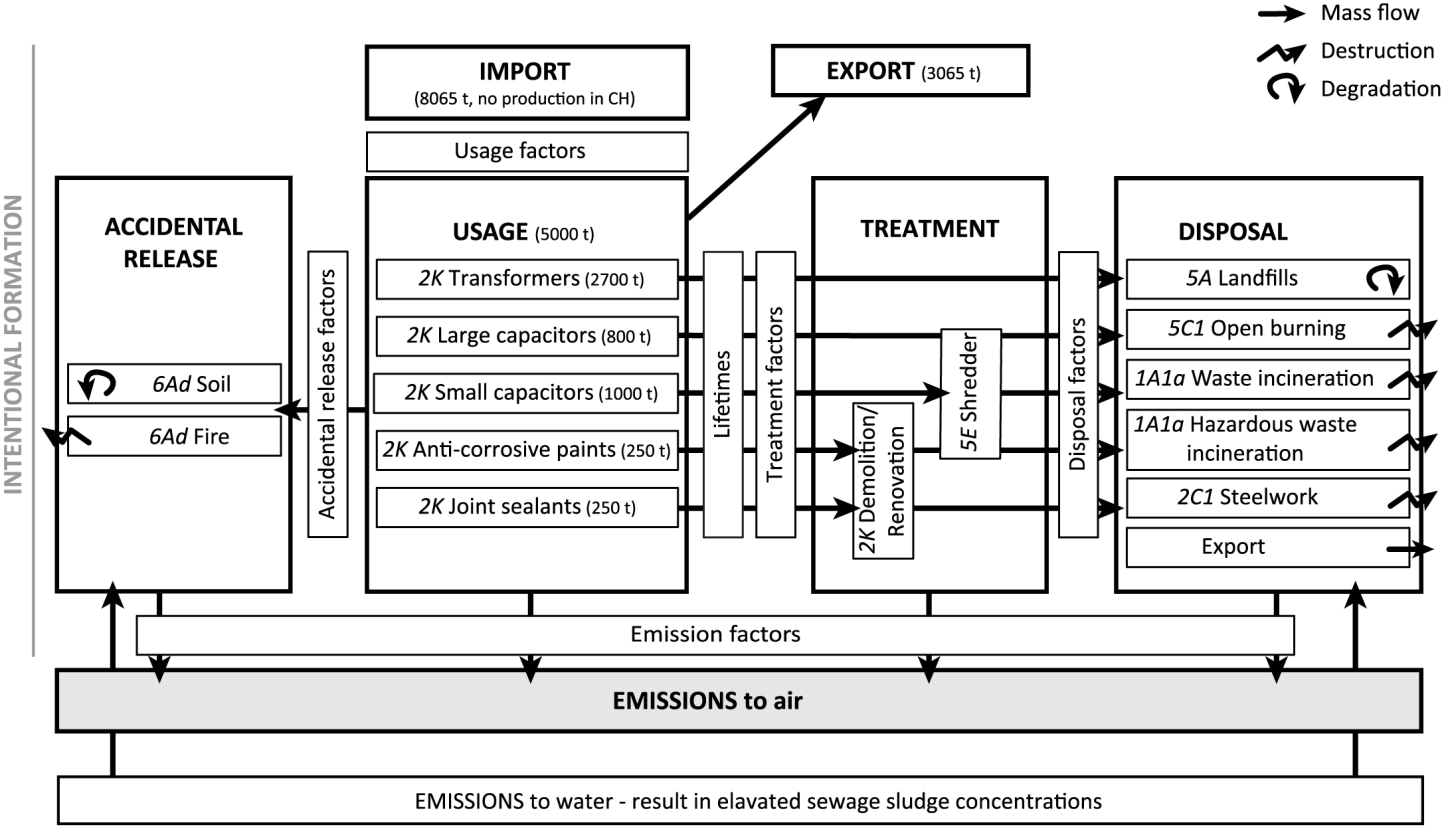
Model setup for the dynamic mass flow and emission model. The imported amount of PCBs is distributed to five usage categories transformers, large capacitors, small capacitors, anti-corrosive paints, and joint sealants. A part of the imported amount is exported in devices. When the used products reach their end of life, they are disposed of to the disposal categories landfills, open burning, waste incineration, hazardous waste incineration, steelworks, and export. For some categories, disposal is preceded by a treatment step. From the usage categories, PCBs can be accidentally released to soil or to fire, or they can enter the waste water and end up in sewage sludge. The model is a dynamic model, running from 1930 to 2100. All disposal factors and most of the emission factors change over time to account for technology improvements. Emissions to air occur from usage, treatment, disposal, and accidental release. The letters in italic are the NFR codes for the CLRTAP emission inventory. Figure adapted from Breivik et al. [15].

The mass balance model tracks the PCBs through their life-cycle of import, usage, and disposal between 1930 and 2100 (Fig 1). The sub-categories defined in this model are also categories in the Swiss Emission Information System (EMIS) in accordance with the UNECE Guidelines for the reporting of emission data under the CLRTAP convention using the nomenclature for reporting (NFR) [26] (italic letters in Fig 1). The PCBs that are imported to Switzerland are distributed to the usage categories and are disposed of to the waste categories after a certain lifespan. Accidental release of PCBs and emissions from sewage sludge treatment are also included. PCB emissions to the environment occur from all stages of their life-cycle. The emissions to air are calculated by multiplying the annual mass of PCBs involved in a process with an emission factor of PCBs from the corresponding process. The five usage categories as well as landfill’ and soils are PCB stocks, which means that PCBs are stored in these categories and passed on through the system with a temporal delay. In these cases, the mass of PCBs involved in the process are the PCBs stored in the stock. The treatment categories of renovation and shredder, the temporary storage category sewage sludge, and the incineration categories (including fire) are instantaneous categories, where PCBs are not stored. In these cases, the mass of PCBs involved in the process are the PCBs treated or incinerated in the same year. The disposal factors and some of the emission factors change over time to account for technology improvements. The presented model is therefore a (deterministic and) dynamic mass flow and emission model.

### Import

#### Import after 1975

**Fig 2:**
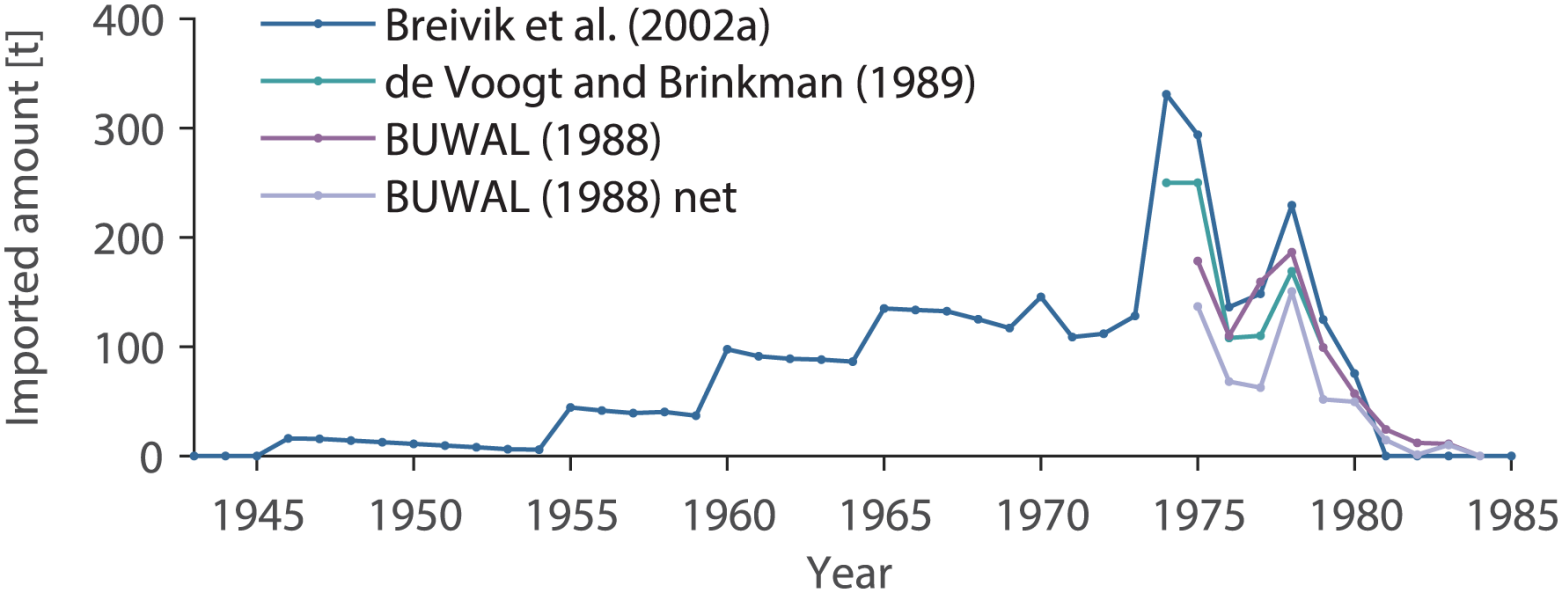
PCB import to Switzerland between 1943 and 1985 as reported in the literature [21, 28, 29]. In total, this data estimate that a mass of 1837 t PCBs was imported and used in Switzerland between 1946 and 1984.

PCBs have not been produced in Switzerland. The chemicals enter the system therefore solely through import (Fig 1, top part). For Switzerland, a national PCB balance, including import, export, usage, and disposal of PCBs is available from 1975 onwards [27, 28]. The PCB import includes pure PCBs and PCBs in devices (Fig 2). The statistics include also the mass of PCBs exported in devices [28]. Averaged over the years 1975 to 1983, 38% of the imported PCBs were exported in devices. The total amount of imported minus exported PCBs (pure PCBs and in devices) between 1975 and 1983 was 545 t.

#### Import until 1975

The study by DeVoogt and Brinkman [29] reports data from the OECD (Organization for Economic Co-operation and Development), an organisation that Switzerland has submitted import data to [27]. The data of Breivik et al. [21] are based on the study of DeVoogt and Brinkman [29]. For the years when no annual report is available, Breivik et al. [21] estimated the PCB import by estimating the production and export from PCB-producing countries and distributed this amount to the receiving countries proportionally based on their gross domestic product (Fig 2, sum of iPCBs multiplied by a factor of 4.6) [21]. The data of Breivik et al. [21] presented here correspond to the high scenario, as this scenario has repeatedly been shown to be the scenario best reproducing measured environmental levels [30–34]. The import increased until the mid-1970s and decreased thereafter. The total amount that was estimated to have been imported between 1930 and 1974 to Switzerland is 2118 t (sum of iPCBs multiplied by a factor of 4.6). If we assume an export of 38%, this number reduces to 1292 t.

### Usage

In this study, five usage categories that were identified to be important for Switzerland are included: transformers, large capacitors (>1 kg), small capacitors (<1 kg), anti-corrosive paints, and joint sealants. The usage category anti-corrosive paints includes paints on steel. Other uses, such as PCBs in hydraulic oils (used in mining), plastics, or insecticides are considered as being of minor importance for Switzerland and are therefore not included in this study.

#### Used amounts

The largest amounts of PCBs were used in Switzerland in transformers. The number of PCB-containing transformers in use in Switzerland in 1986 was reported to be 2000–3000, containing in total approximately 1800–2700 t PCBs [28]. The mass of PCBs in use in transformers in the years before 1986 might have been even higher, because the usage of PCBs in closed applications was banned in 1986. We therefore used the upper bound of the reported amount.

The reported number of large PCB-containing capacitors in use in 1986 was 5000–7000, containing 1200–1800 t of PCBs [28]. We think that the upper bound should be lower since the PCB content per capacitor would be unrealistically high, and because some large capacitors were only partially PCB contaminated. An extrapolation of a study done in the Canton of Aargau to the whole of Switzerland estimates an amount of 800 t of PCBs used in large capacitors [35, 36]. This amount was also used in this study.

The mass of PCBs used in small capacitors in the Federal Republic of Germany was estimated as 10’000 t [37]. Using a per-capita approach and the population sizes at that time (Germany 62 million inhabitants, Switzerland 6.7 million inhabitants) results in 1000 t PCBs used in small capacitors in Switzerland. This number agrees well with the 540 t PCBs used solely in fluorescent light ballasts in Switzerland, as estimated by Kuhn and Arnet [38].

The PCB amounts used in anti-corrosive paints and joint sealants in Switzerland were estimated to be 150–300 t [25] and 100–300 t [39], respectively. In comparison, the study by Kohler et al. [40] estimated the mass of PCBs still present in joint sealants in 2005 to be 50–150 t.

**Fig 3:**
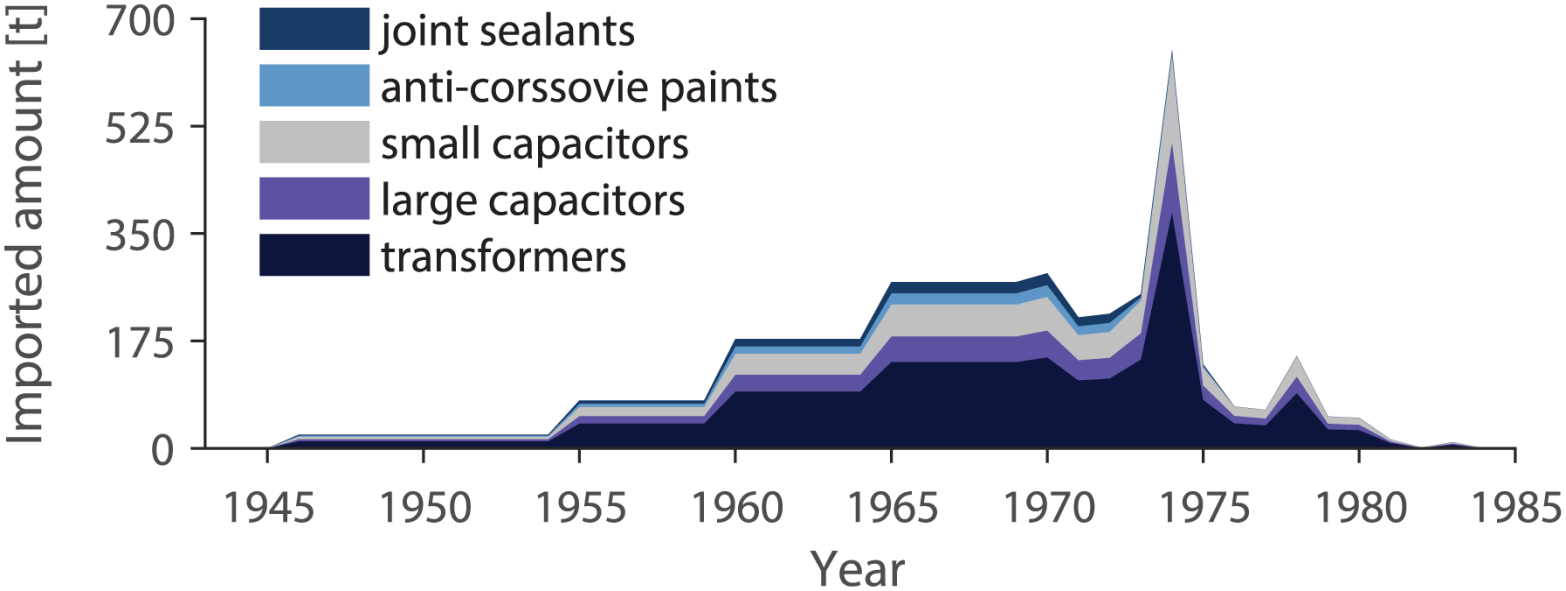
Annually new Swiss PCB consumption in the five usage categories transformers, large capacitors, small capacitors, anti-corrosive paints, and joint sealants between 1940 and 1985. The total masses of PCBs used in the five categories are set to 2700, 800, 1000, 250, and 250 t, respectively. The PCB consumption shown here is the product of the imported amount and the usage factors for every year.

The precise PCB masses used in Switzerland are not known. The Swiss Federal Office for the Environment, Forests, and Landscape (BUWAL) stated in 2000 that 6000 t of PCBs were used in Switzerland between 1930 and 1980. They assumed that 4000 of the 6000 t have been used in closed applications (transfomers, large capacitors) and around 2000 t in open applications (small capacitors, plasticisers, paints, joint sealants, flame retardants, etc.) [25]. According to the reported data, the net registered PCB import was 1837 t. In contrast, PCB usage in the five usage categories was estimated to be higher than this number. Hence, in this study, we use total usages of 2700, 800, 1000, 250, and 250 t for the categories transformers, large capacitors, small capacitors, anti-corrosive paints, and joint sealants, respectively (Fig 3). This results in a total mass of 5000 t of PCBs used. Considering 38% export of PCBs in devices, Switzerland must have imported around 8000 t of PCBs.

#### Temporal trend

To retain the temporal trends of the PCBs imported, we applied for the PCBs in use: i) the data of Breivik et al. [21] for the years 1930 to 1974 minus an export of 38% times a factor of 3.45. This factor corresponds to the quotient of the estimated used amount of PCBs in Switzerland between 1930 and 1974 (4455 t) and the estimated imported amount of PCBs between 1930 and 1974 (1292 t). ii) For the years 1975–84, the net BUWAL data is used [28], since in Switzerland there were special permissions for using PCBs in capacitors of fluorescent light ballasts until 1983 [41]. The transient decreases in import starting in 1945, 1955, 1960, and 1965 in the data of Breivik et al. [21] are caused by the uniform distribution of production data reported for 5- or 10-year periods. Here, this artefact is removed by averaging the data over these time periods (i.e. 1946–1954, 1955–1959, 1960–1964, and 1965–1969) (see Fig 3).

#### Usage factors

For every year, the imported amount of PCBs is distributed to the five usage categories by usage factors. These factors are calculated relative to the total PCB consumption in the five categories. For this, three periods are identified: 1946–72: closed and open applications; 1973–75: linearly decreasing open applications; 1976–83: only closed applications. For example, the PCB amount imported in 1976 is distributed to the three categories transformers, large capacitors, and small capacitors using usage factors of 0.6, 0.18, and 0.22, which are derived from the total import mass of these three categories (2700, 800, and 1000 t, respectively). The PCB consumption in a specific year and category is then calculated by multiplying the usage factor with the imported amount (Fig 3).

### Lifespans and disposal

#### Calculation of lifespans

The lifespans are very sensitive parameters in this inventory and an increase or decrease of the lifespans of only a view years can change the results quite substantial. It is therefore important to define the lifespans as accurate as possible. The average lifespans of the five usage categories in this inventory are defined according to three sets of information: i) the reported mass of PCBs in use in Switzerland in a specific category and a specific year, ii) the reported mass of PCBs disposed of in Switzerland from a specific category in a specific year, and iii) the lifespans of the products in the usage categories reported in literature (Section B in Emissions). Based on these information sets, we estimated the lifespans of transformers and large capacitors to be 25 years, the lifespans of small capacitors to be 22 years, and the lifespans of anti-corrosive paints and joint sealants to be 55 years.

#### Weibull distribution

**Fig 4:**
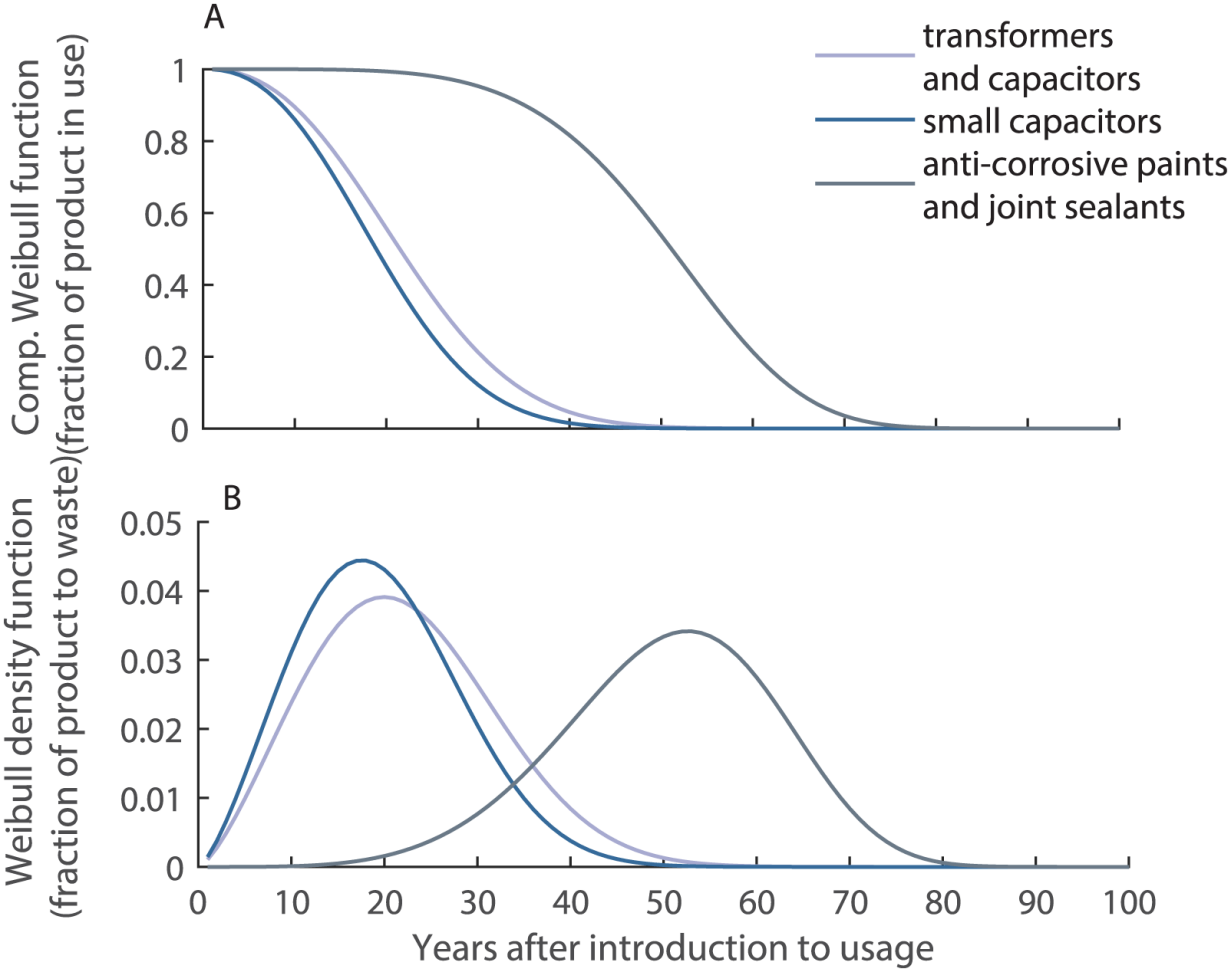
Complementary Weibull function and Weibull density function. A: The complementary Weibull function describes the fraction of product in use in the years after the introduction of the product to the use phase. B: The Weibull density function describes the fraction of product disposed of in every year after the introduction of the product to the use phase.

The disposal of a product as a function of its average lifespan is calculated by a Weibull distribution. The Weibull distribution describes the probability that a product is disposed of in the years after the product enters the use phase. This distribution has been used in comparable studies before, for instance to describe the stocks and flows of products containing the flame retardants polybrominated diphenyl ethers (PBDEs) [42]. The complementary Weibull function *W*′(*t*) describes the fraction of product in use and is a function of the average lifespan *T* of a product type, a distribution parameter *k* (unitless, also called a shape parameter), the point in time the product enters the use phase *t*_0_, and the usage time *t* (Eq (2), Fig 4A).

$$\begin{array}{c} W^{\prime }\left(t\right)=\exp^{-{\left(\frac{t-t_{0}}{T}\right)}^{k}} \end{array}$$ (2)

For every year after the introduction of a product in the usage phase, the fraction of product disposed of as waste relative to the fraction still in use, is determined by the Weibull density function *w(t)* (Eq (3), Fig 4B).

$$\begin{array}{c} w\left(t\right)=\frac{k}{T}\cdot {\left(\frac{t-t_{0}}{T}\right)}^{k-1}\cdot \exp^{-{\left(\frac{t-t_{0}}{T}\right)}^{k}} \end{array}$$ (3)

The shape parameter *k* can be set to 2.4 for electronic devices (transformers and capacitors) [42] and to 5.0 for steel constructions (anti-corrosive paints) [43]. For joint sealants, the value of anti-corrosive paints is used. A low shape parameter leads to a disposal of the product over a long time period (dissimilar lifetimes), while a high parameter induces disposal over a short time period (more similar lifetimes).

#### Disposal categories and disposal factors

In this study, six disposal categories that have been historically relevant in Switzerland are included: landfills, open burning, municipal waste incineration, hazardous waste incineration, steelworks, and export (Fig 1, right part). Here, open burning represents outdoor fires that are not in a container. Steelworks represents scrap metal that is melted and thereby PCB-containing paint residues are combusted in furnaces at temperatures of around 1600°C. Landfills are disposal sites where the waste is dumped. In landfills, PCBs are partly stored and partly degraded. When waste is exported, emissions abroad are not included in the Swiss emission inventory. The allocation of the different products to the different waste processes (expressed by disposal factors) changes over time to account for technology improvements. The disposal factors are given in Table 2, the disposal routes of the PCB-containing products are described in detail in Section C in S1 File.

**Table 2:** Swiss-specific time-dependent disposal factors for the disposal of transformers, large capacitors, small capacitors, anti-corrosive paints, joint sealants, and sewage sludge used in the model. Factors for years which are not mentioned explicitly are linearly interpolated. Before disposal, PCBs used in anti-corrosive paints and joint sealants undergo treatment by renovation/demolition. This process is not mentioned here explicitly, as the total amount of PCBs contained in these categories undergoes this treatment step.

year	landfill	open burning	waste incineration	export	shredder landfill	shredder waste incineration	hazardous waste incineration	steelwork	agricultural fertilizer
**transformer and large capacitors**									
1940	0.80	0.10	0.10	-	-	-	-	-	-
1972	0.56	0.10	0.31	0.03	-	-	-	-	-
1980	0.28	0.10	0.36	0.26	-	-	-	-	-
1991	-	-	0.43	0.57	-	-	-	-	-
1998	-	-	0.27	0.67	-	-	0.06	-	-
1999	-	-	0.25	0.24	-	-	0.51	-	-
2000	-	-	0.23	0.21	-	-	0.56	-	-
2010	-	-	-	0.21	-	-	0.76	-	-
**small capacitors**									
1940	0.60	0.20	0.20	-	-	-	-	-	-
1969	0.47	-	0.53	-	-	-	-	-	-
1972	0.37	-	0.60	0.03	-	-	-	-	-
1978	0.12	-	0.68	0.20	-	-	-	-	-
1998	-	-	0.10	0.67	0.16	-	0.07	-	-
1999	-	-	0.10	0.24	-	0.59	0.07	-	-
2000	-	-	0.10	0.16	-	0.67	0.07	-	-
2010	-	-	0.01	0.16	-	0.21	0.62	-	-
**anti-corrosive paints**									
1940	0.90	-	-	-	-	-	-	0.10	-
1990	0.10	-	0.80	-	-	-	-	0.10	-
1995	0.05	-	0.15	-	-	-	0.75	0.05	-
2012	0.03	-	0.09	0.10	-	-	0.75	0.03	-
**joint sealants**									
1940	0.90	0.10	-	-	-	-	-	-	-
1995	0.60	0.10	0.30	-	-	-	-	-	-
2003	0.10	-	0.10	-	-	-	0.80	-	-
**sewage sludge**									
1974	0.20	-	-	-	-	-	-	-	0.80
1980	0.34	-	0.01	-	-	-	-	-	0.65
1984	0.45	-	0.05	-	-	-	-	-	0.50
1989	0.39	-	0.11	-	-	-	-	-	0.50
1994	0.28	-	0.17	-	-	-	-	-	0.55
2000	0.02	-	0.59	0.01	-	-	-	-	0.38
2002	0.02	-	0.71	0.06	-	-	-	-	0.21
2006	-	-	0.94	0.06	-	-	-	-	-

### Treatment

Before disposal, some usage categories undergo specific treatment processes (Fig 1, right part). Two treatment categories are included in this inventory: 1) demolition/renovation of steel constructions (containing PCBs in anti-corrosive paints) and buildings (containing PCBs in joint sealants), and 2) shredding of electronic waste (with PCBs in small capacitors). Demolition/renovation can induce elevated emissions to the environment, as has been observed for buildings [44]. Shredding of electronic waste occurs at a fast rotation velocity that leads to an increased temperature and dust production [45]. Shearing of steel constructions (heavy scrap) is otherwise assumed to produce little dust with no evaporation of the substances in the coating [45]. We assume therefore no emissions to air from the shearing of steel constructions.

### Accidental release

From each usage category, PCBs can be accidentally released (Fig 1, left part). Here, this release includes two categories: soil and fire. When released to soil, PCBs are partly stored and partly degraded. In the case of fire, PCBs are partly destroyed by high temperatures and partly emitted to the environment. The release factors from the use categories to soil and fire are shown in Table 3. A detailed description is provided in Section D in S1 File.

**Table 3:** Accidental release factors for the usage categories. The factors are the same for all PCB congeners. The units are in weight per weight per year (1/year). For references please refer to Section C in S1 File.

factor	all congeners
transformers to soil	0.0001
large and small capacitors to soil	0.002
anti-corrosive paints and joint sealants to soil	0.002
all usage categories to fires	0.001

### Degradation

For degradation in soil and landfills, the values reported by Bogdal et al. [2] for 25°C are adapted to a temperature of 10°C (Table 4). The values are based on degradation half-lives presented in the book by Mackay [46]. The degradation rates decrease with an increasing level of chlorination.

**Table 4:** Degradation rate constants and emission factors for usage, treatment, disposal, and accidental release for the six indicator PCB congeners. The emission factors are in weight per weight per year, the degradation rates are ‘per year’. Emission factors for landfills between 1980 and 2050, for the renovation of joint sealants between 1999 and 2007, and the renovation of anti-corrosive paints between 1998 and 2006 were interpolated linearly. Emission factors for municipal waste incineration, hazardous waste incineration, and steelworks for years not mentioned in the table were extrapolated according to the PM 2.5 emission factors of the respective industrial plant. For references and derivations please refer to the text and Section E in S1 File.

PCB congener	PCB-28	PCB-52	PCB-101	PCB-138	PCB-153	PCB-180
**emissions from usage**						
transformers	2.36 ⋅ 10^−5^	1.29 ⋅ 10^−5^	2.58 ⋅ 10^−6^	2.14 ⋅ 10^−7^	4.18 ⋅ 10^−7^	2.76 ⋅ 10^−8^
large and small capacitors	5.90 ⋅ 10^−4^	3.24 ⋅ 10^−4^	6.45 ⋅ 10^−5^	5.35 ⋅ 10^−6^	1.05 ⋅ 10^−5^	6.90 ⋅ 10^−7^
joint sealants	1.35 ⋅ 10^−2^	8.06 ⋅ 10^−3^	2.20 ⋅ 10^−3^	4.62 ⋅ 10^−4^	5.84 ⋅ 10^−4^	1.49 ⋅ 10^−4^
anti-corrosive paints	6.75 ⋅ 10^−2^	4.03 ⋅ 10^−2^	1.10 ⋅ 10^−2^	2.31 ⋅ 10^−3^	2.92 ⋅ 10^−3^	7.45 ⋅ 10^−4^
**emissions from treatment**						
shredders	8.65 ⋅ 10^−2^	5.63 ⋅ 10^−2^	1.45 ⋅ 10^−2^	7.46 ⋅ 10^−3^	6.52 ⋅ 10^−3^	4.91 ⋅ 10^−3^
renovation of buildings (joint sealants) (1930–1999)	1.35 ⋅ 10^−1^	8.06 ⋅ 10^−2^	2.20 ⋅ 10^−2^	4.62 ⋅ 10^−3^	5.84 ⋅ 10^−3^	1.49 ⋅ 10^−3^
renovation of buildings (joint sealants) (2007–2100)	1.35 ⋅ 10^−2^	8.06 ⋅ 10^−3^	2.20 ⋅ 10^−3^	4.62 ⋅ 10^−4^	5.84 ⋅ 10^−4^	1.49 ⋅ 10^−4^
renovation of steel constructions (anti-corrosive paints) (1930–1998)	6.75 ⋅ 10^−1^	4.03 ⋅ 10^−1^	1.10 ⋅ 10^−1^	2.31 ⋅ 10^−2^	2.92 ⋅ 10^−2^	7.45 ⋅ 10^−3^
renovation of steel constructions (anti-corrosive paints) (2006–2100)	6.75 ⋅ 10^−2^	4.03 ⋅ 10^−2^	1.10 ⋅ 10^−2^	2.31 ⋅ 10^−3^	2.92 ⋅ 10^−3^	7.45 ⋅ 10^−4^
**emissions from disposal**						
landfills (1930–1980)	2.98 ⋅ 10^−4^	2.63 ⋅ 10^−4^	5.45 ⋅ 10^−5^	3.37 ⋅ 10^−5^	1.57 ⋅ 10^−5^	3.58 ⋅ 10^−6^
landfills (2050–2100)	2.98 ⋅ 10^−6^	2.63 ⋅ 10^−6^	5.45 ⋅ 10^−7^	3.37 ⋅ 10^−7^	1.57 ⋅ 10^−7^	3.58 ⋅ 10^−8^
open burning	0.10	0.10	0.10	0.10	0.10	0.10
municipal waste incineration (1996)	2.28 ⋅ 10^−3^	3.18 ⋅ 10^−3^	1.32 ⋅ 10^−3^	6.70 ⋅ 10^−4^	6.70 ⋅ 10^−4^	6.50 ⋅ 10^−4^
hazardous waste incineration (1998)	1.00 ⋅ 10^−6^	1.00 ⋅ 10^−6^	1.00 ⋅ 10^−6^	1.00 ⋅ 10^−6^	1.00 ⋅ 10^−6^	1.00 ⋅ 10^−6^
steelworks (2014)	3.42 ⋅ 10^−2^	4.77 ⋅ 10^−2^	1.98 ⋅ 10^−2^	1.01 ⋅ 10^−2^	1.01 ⋅ 10^−2^	9.75 ⋅ 10^−3^
export	-	-	-	-	-	-
**emissions from accidental release**						
soil	3.36 ⋅ 10^−3^	1.91 ⋅ 10^−3^	6.98 ⋅ 10^−4^	2.26 ⋅ 10^−4^	2.74 ⋅ 10^−4^	6.98 ⋅ 10^−5^
fires	0.10	0.10	0.10	0.10	0.10	0.10
**degradation rate constants in soil and landfills**	0.208	0.123	0.021	0.004	0.004	0.002

### Emissions to air

Emissions to air occur from the entire system: usage, treatment, disposal, and accidental release (Fig 1, gray shaded area). The emissions are calculated by multiplying the annual mass of PCBs involved in a process with the emission factor for the corresponding process. The emission factors are defined as mass of PCBs emitted per mass of PCBs in a category. The reason why the factors are a function of the mass of PCBs and not a function of the mass of product (for example mass of waste or amount of air) is that the PCB content is strongly variable between the products and with time. Table 4 shows the emission factors for emissions from usage, disposal, treatment, and accidental release. The emission factors for renovation as well as for municipal waste incineration, hazardous waste incineration, steelworks, and landfills are time variable to account for the technological improvements over time; the other emission factors are constant over time. A detailed description of the used factors and additional explanations are provided in Section E in S1 File. Emissions to air from unintentional formation are not included in the inventory. We assume, based on data from the FOEN [12], that unintentional formation was unimportant for Switzerland between 1980 and 2014; however, it might have been more important before 1980. The exclusion of unintentional formation therefore represents an intrinsic uncertainty to the inventory between 1930 and 1980 that is clearly noted.

### Emissions to water bodies

The release of PCBs to water bodies is only partly included in this study. Release to water bodies is important for anti-corrosive paints and to a smaller degree also for leachate from landfills [47]. However, emission factors for the release of PCBs from anti-corrosive paints and landfills to water are missing. Additionally, other sources, which are not included in this study such as recycled toilet paper [48], may also cause emissions to the hydrosphere. To be able to quantify these emissions at all, we decided to treat emissions to water bodies differently to all other emissions. Instead of using emission factors from the use and disposal categories, we used measured PCB concentrations in sewage sludge and the total amount of produced sewage sludge per year to determine the mass of PCBs released to water. This approach overlooks emissions to natural water bodies, but it captures emissions to waste water. The model considers four disposal pathways for sewage sludge that have been historically relevant in Switzerland: usage as fertilizer in agriculture, dumping to landfills, incineration in municipal or sludge incineration plants or in cement work, and export (Table 2). More information is provided in Section F in S1 File.

### Monte carlo analysis

Uncertainties in the model were calculated using a Monte Carlo simulation. For this, we defined a distribution function and an uncertainty factor for all parameters. This uncertainty factor was the confidence factor [49] for log-normally distributed parameters or the standard deviation for normally distributed parameters (Section G in S1 File). The simulations where performed using Latin hypercube sampling and a sampling size of 250 [50].

## Results

### PCB amounts in the life-cycle of usage, storage, treatment and disposal

#### Used amounts

**Fig 5:**
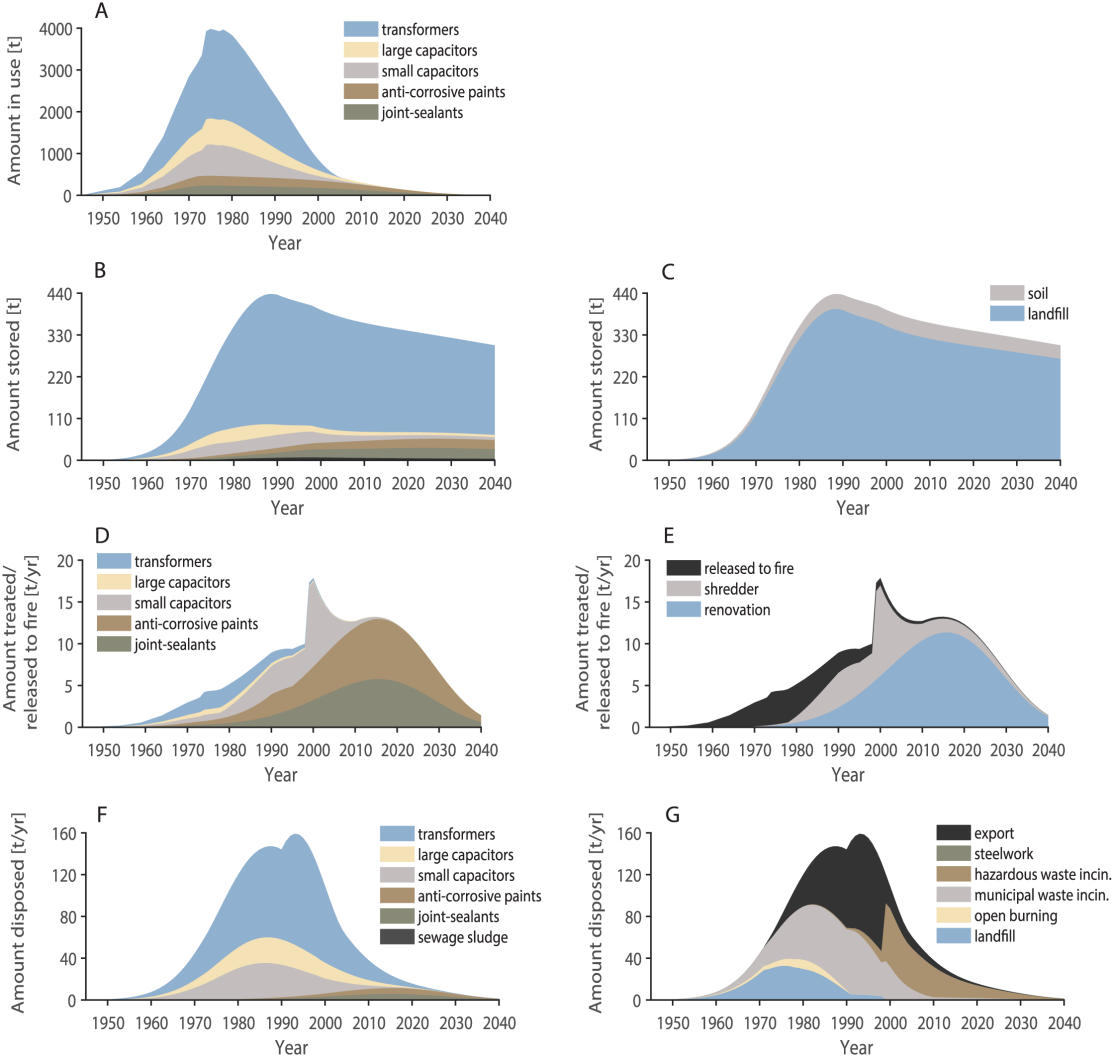
Swiss PCB amounts in the life-cycle of usage, storage, treatment and disposal between 1945 and 2040. The amounts in sub-figure A) to C) are stocks and depend on the used/stored amount of the previous years. The amounts in sub-figure D) to G) are by contrast yearly treated/disposed amounts which are independent of the previously treated or disposed amounts. The legend in sub-figure B is the same as in F. A: PCB amounts in use. B: PCB amounts stored, subdivided in use categories. C: PCB amounts stored, subdivided in storage categories. D: PCB amounts treated/released accidentally to fire, subdivided in use categories. E: PCB amounts treated/released accidentally to fire, subdivided in treatment categories. F: PCB amounts disposed of, subdivided in use categories. G: PCB amounts disposed of, subdivided in disposal categories.

PCBs were used in Switzerland from 1946 onwards and they are still present in many applications today. The usage peaked in 1975 at around 4000 t of PCBs (Fig 5A). 2140 of these 4000 t in 1975 were PCBs used in transformers, and 620, 750, 240, and 240 t were PCBs in use in large capacitors, small capacitors, anti-corrosive paints and joint sealants, respectively. According to our model, 217 t of PCBs are still in use in all applications in Switzerland today. 33 of these 217 t in 2017 are PCBs used in transformers, and 9, 3, 86, and 86 t are PCBs in use in large capacitors, small capacitors, anti-corrosive paints and joint sealants, respectively. The share of PCBs in transformers relative to the total amount of PCBs in use in a specific year thus decreased from 54% in 1975 to 15% in 2017 due to the shorter life-time and faster disposal of PCBs in transformers compared to PCBs in anti-corrosive paints and joint sealants.

#### Stored amounts

Landfills and soils are PCB stocks, which means that PCBs are stored in these categories and removed from the system with a temporal delay. According to our model, the highest PCB amounts were stored in landfills in 1989 (400 t) and have decreased slowly since then (Fig 5B and 5C). We calculated that around 300 t of PCBs are still stored in landfills today and that this amount will only decrease to 250 t in 2050 and to 200 t in 2100. The amount of PCBs in soil peaked in 2000 at around 42 t. Again, this amount will only decrease very slowly. We calculated that 33 t will still be stored in soil in 2050 and 24 t in 2100. The amount of PCBs stored in soil that originated from sewage sludge was in all years below 10%.

#### Treated/accidental released amounts

The two treatment categories included in this inventory are demolition/renovation of steel constructions (containing PCBs in anti-corrosive paints) and buildings (containing PCBs in joint sealants) as well as shredding of electronic waste (with PCBs in small capacitors). The treated PCB amounts in demolition/renovation peaked in 2016, the amounts in shredders peaked in 2000, both at 11 t PCBs per year (Fig 5D and 5E). The amounts of PCBs accidentally released to fire were much smaller and peaked in 1975 at 4 t PCB per year.

#### Disposed amounts

PCBs in Switzerland were disposed of to landfills, open burning, municipal waste incineration, hazardous waste incineration, and to steelworks. Parts were also exported. Landfilling was important until the late 1980s and decreased thereafter (Fig 5G). Municipal waste incineration and export were the main disposal pathways between the mid-1980s and the year 2000, thereafter they were replaced by hazardous waste incineration. The disposal pattern was determined by the disposal of transformers, whereas anti-corrosive paints and joint sealants played minor roles (Fig 5F). The peaks between 1990 and 2000 originate from changes in waste disposal in that time period, for example the transformation from disposing shredder residues (small capacitors) in landfills to treating them in waste incinerators. Detailed disposal patterns for the individual disposal pathways are provided in Section H in S1 File.

### Emissions to air

#### Total emissions

**Fig 6:**
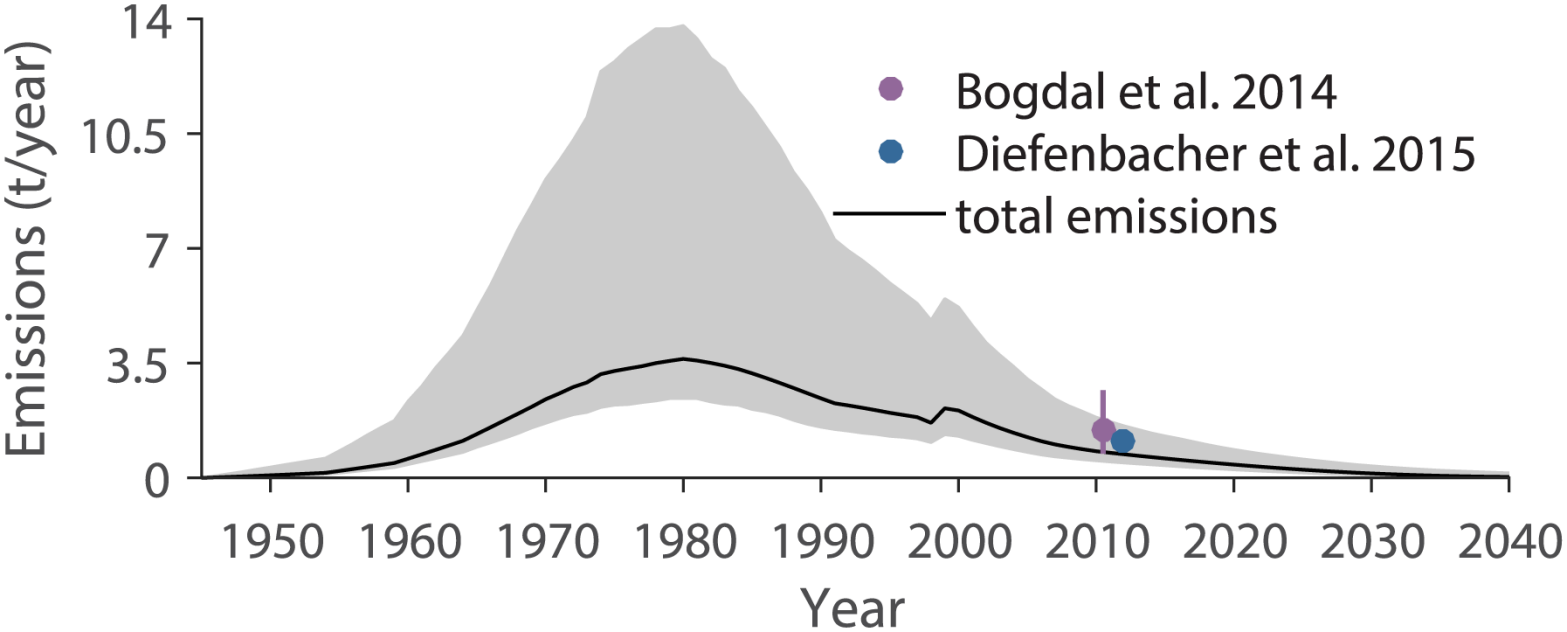
Total Swiss PCB emissions to air from all categories (black line) with their uncertainty ranges (10^th^ and 90^th^ percentile) in gray. The emissions estimated in the studies of Bogdal et al. [51] and Diefenbacher et al. [52] are shown for comparison. The bars represent the uncertainties in the measurements.

**Fig 7:**
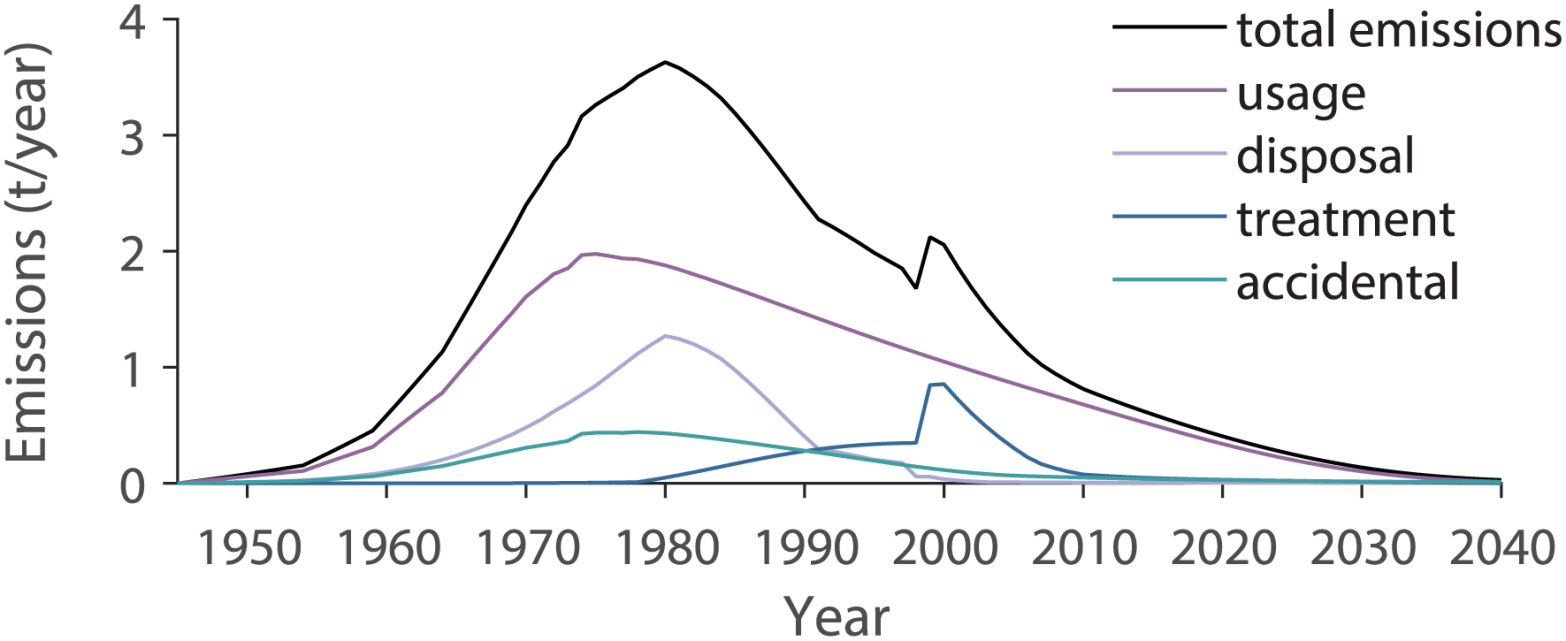
Total Swiss PCB emissions to air and the sums of the emissions from the categories usage, disposal, treatment, and accidental release between 1945 and 2040.

**Fig 8:**
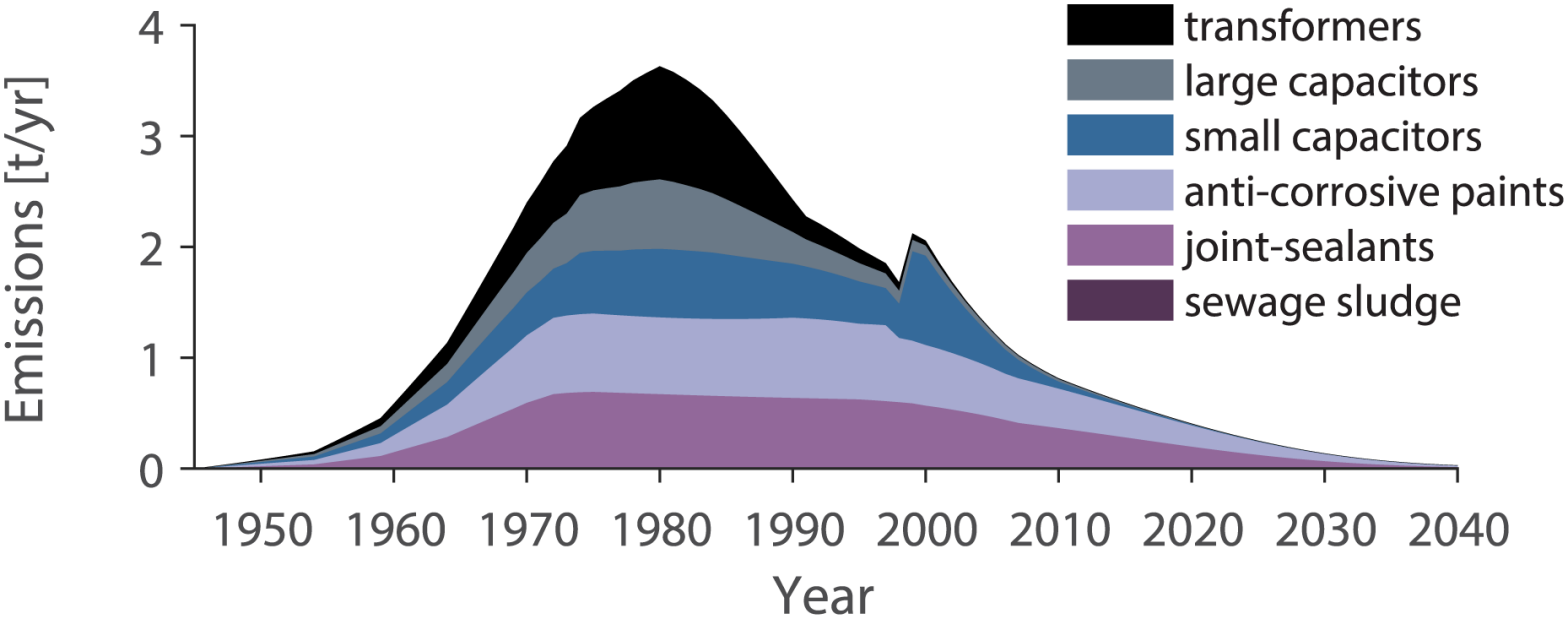
Total Swiss PCB emissions to air divided into emissions from transformers, large capacitors, small capacitors, anti-corrosive paints, joint sealants, and sewage sludge between 1945 and 2040.

Total PCB emissions to air from all categories calculated by the model increase until the 1980s and reach a peak value of 3.6 t PCBs/year in 1980 (black line in Fig 6). Thereafter, the emissions decrease to a low level. Considering the uncertainties in the parameters, we estimate that emissions to air in 1980 were in the range from 2.4 to 14 t PCBs/year. Today, the emissions decrease continuously, with a decrease of 7% between 2016 and 2017. The total emissions to air and the sums of the emissions from the categories usage, disposal, treatment, and accidental release are shown in Fig 7. Emissions from usage were the most important emissions during the whole time period. The total emissions to air divided according to the use categories plus emissions from the use and disposal of sewage sludge are shown in Fig 8. Anti-corrosive paints and joint sealants were the most important PCB emission sources until 1974, between 1986 and 1998, and after 2002. Emissions from transformers were highest between 1975 and 1985, and emissions from small capacitors were highest between 1999 and 2002. Emissions to air from the usage and disposal of PCB-containing sewage sludge were negligible compared to the other emission sources.

#### Emissions from individual categories

**Fig 9:**
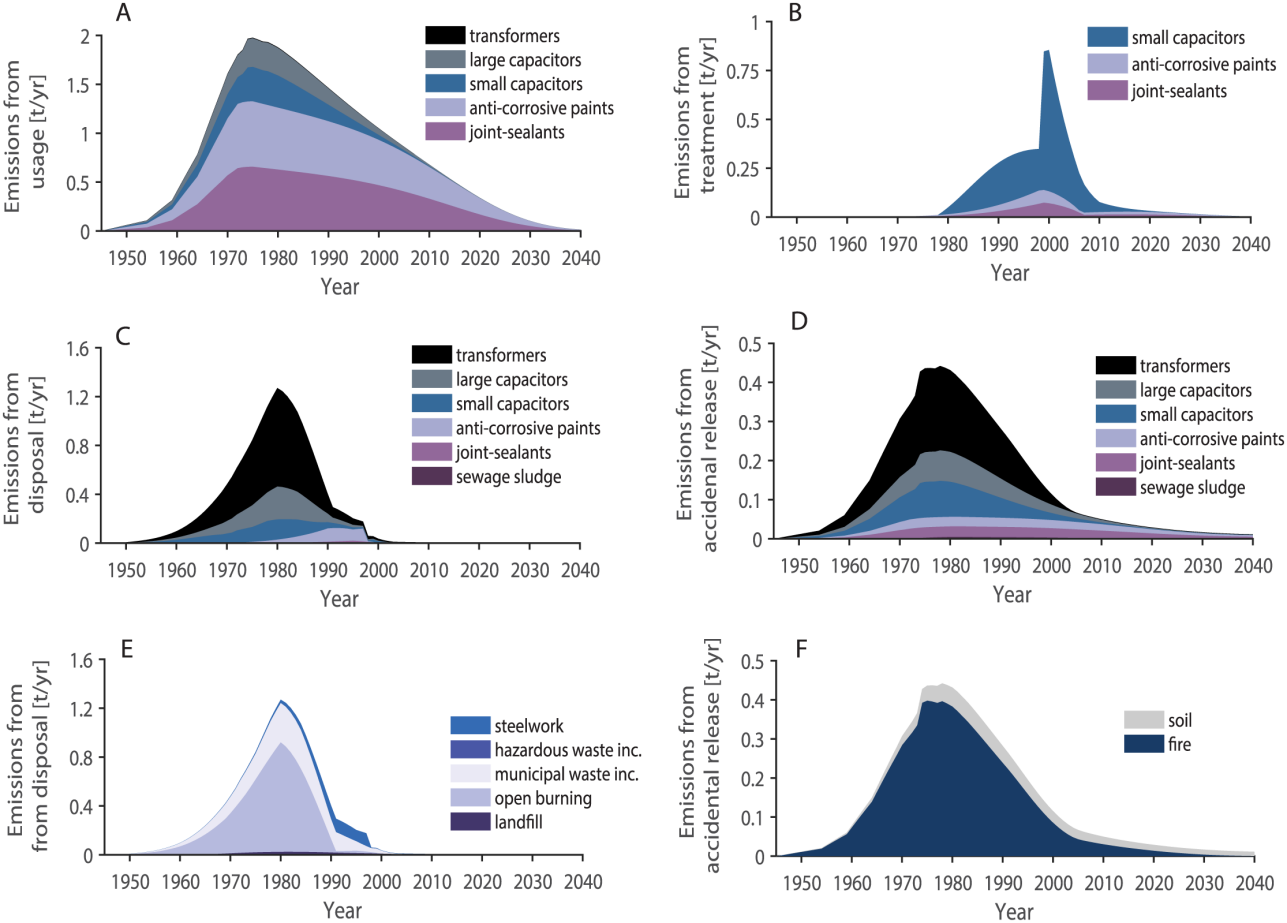
Total Swiss PCB emissions to air from the single categories between 1945 and 2040. A: Emissions from the usage categories. B: Emissions from treatment. C: Emissions from disposal, subdivided in use categories. D: Emissions from accidental release, subdivided in use categories. E: Emissions from disposal, subdivided in disposal categories. F: Emissions from accidental release, subdivided in accidental release categories.

Emissions to air from use increased throughout the 1970s and reached a peak value of 2.0 t PCBs/year in 1975 (Fig 9A). Considering the uncertainties in the parameters, we estimate that emissions in 1975 were in the range of 1.2 to 6.3 t PCBs/year. The largest contributions came from the PCB usage in anti-corrosive paints and joint sealants, followed by large and small capacitors. Emission from the use in transformers are of minor importance, although the largest PCB amounts were used in this category (see also Table 5).

**Table 5:** Emissions to air in Switzerland from 1930 to 2017 in [t] from transformers, large capacitors, small capacitors, anti-corrosive paints, joint sealants, and sewage sludge.

	Emissions to air from 1930 to 2017 in [t] from					
	transformers	large capacitors	small capacitors	anti-corrosive paints	joints sealants	sewage sludge
usage	0.123	7.95	8.81	29.5	29.5	0
release from fire	5.51	1.69	1.88	1.02	1.02	0
release from soil	0.060	0.525	0.585	0.125	0.255	0.15
landfilling	0.358	0.179	0.216	0.001	0.013	0.006
open burning	10.5	3.01	0.181	0	0.212	0
municipal waste inc.	2.36	1.40	2.96	0.016	0.015	0.005
hazardous waste inc.	1.86 ⋅ 10^−4^	4.22 ⋅ 10^−5^	6.06 ⋅ 10^−5^	3.21 ⋅ 10^−5^	2.31 ⋅ 10^−5^	0
steelworks	0	0	0	1.54	0	0
renovation	0	0	0	1.12	1.22	0
shredding	0	0	7.49	0	0	0
**sum**	**18.9**	**14.8**	**22.1**	**33.3**	**32.2**	**0.17**

Emissions from treatment peaked later than the other emission categories, with highest values being reached near 2000 (Fig 9B). This is caused by the late disposal of the open usage categories that have very long lifespans. Considering the uncertainties in the parameters, we estimate that the emissions of 0.9 t PCBs/year to air in 2000 were in the range of 0.5 to 1.7 t PCB/year. The largest part of the emissions originated from shredding of small capacitors.

Emissions from disposal were highest before the 1980s, a feature that is governed by the high emissions of the open burning of transformers and large capacitors (Fig 9C and 9E). Emissions from disposal reached a peak value of 1.3 t PCBs/year in 1980. Considering the uncertainties in the parameters, we estimate that emissions in 1980 were in the range of 0.9 to 4.1 t PCBs/ year. The melting down of steel containing anti-corrosive paints produced the highest emissions between 1988 and 1998. After this, emissions from waste incineration of small capacitors and transformers took over the role of the principal emitter of the disposal categories, with emissions being low but continuous. As discussed in Section E in S1 File, the emission factors of landfills reported in the literature varied over several orders of magnitude, which means that the emissions from landfills reported here, might be over- or underestimated. The contribution of the different use categories to the emissions of the different disposal categories is shown in Table 5 and in Fig I1 in S1 File.

Emissions from accidental release are lower than emissions from the other categories (Fig 9D and 9F, Table 5). Considering the uncertainties in the parameters, we estimate that the peak emissions of 0.44 t PCBs/year in 1975 were in the range of 0.37 to 3.8 t PCB/ year. Until 2020, PCBs will be still in use and emissions from fire will remain important. In contrast, emissions from soil seem negligible during this period. However, after 2040, when emissions from the other categories decrease, emissions from soil persist and will become the most important emission source for emissions to air. Furthermore, long-term emissions from soil might be even more important than they are in this model, since deposition from the atmosphere to soil and subsequent revolatilization is not included in the model (so-called secondary environmental emissions of PCBs).

#### Future emissions

In the model, total emissions to air currently decrease and are predicted to decrease further in the future. In 1990, total PCB emissions to air were above 2.4 t/year, while in 2015, they were near 0.6 t/year. In 2030 atmospheric emissions are predicted to decrease to 140 kg/year. Emissions from usage and treatment were most important in 2015, as the usage and disposal of open applications was still ongoing. This will continue to change until 2050, when emissions from accidental release will be responsible for 92% of the emissions to air, and emissions from use will only account for 6%. Emissions from accidental release from soil and fire will remain the most important emission source for emissions to air in 2100 and will account for 99% of the emissions, while emissions from disposal will be responsible for less than 1% of the emissions. This shows that soils function as long term reservoirs and will emit PCBs also long after their use phase.

#### Emissions in TEQ

The emissions of PCBs can also be expressed in TEQ. Using WHO 1998 TEF values, total PCB emissions to air between 1930 and 2016 have been 1.53 kg WHO(1998)-TEQ. Applying the WHO 2005 TEF values, this corresponds to 0.73 kg WHO(2005)-TEQ. The largest contributions to the WHO(1998)-TEQ value came from PCB-118, -126, and -156, while the largest contributions to the WHO(2005)-TEQ value came from PCB-126, -118, and -105.

## Discussion

### Used amounts

The numbers reported in literature for the PCB use in Switzerland are difficult to verify because the main PCB use phase was 40 years ago. However, PCBs were also used in other countries, so we compare our data to data from Germany to verify the order of magnitude of the reported amounts in Switzerland. The available data indicate that around 23’000 t of PCBs were used in Germany in transformers until 1984 [48]. An extrapolation of this amount (77.8 million inhabitants in Germany in 1984, 6.4 million in Switzerland) suggests the use of 1900 t of PCB in transformers in Switzerland. This amount is a factor of 1.4 smaller than the used amount of 2700 t in this study.

Around 13’000 t of PCBs were used in Germany in large capacitors [48]. The extrapolations give an amount of 1100 t of PCBs in large capacitors in Switzerland. The value used in this study for Switzerland is 800 t and therefore slightly smaller than the extrapolated value.

The amounts of PCBs used in small capacitors in this study were extrapolated from the amounts used in Germany. It is therefore not possible to make a comparison.

Around 24’000 t of PCBs were used in Germany until 1972 in open applications [48]. An extrapolation of this amount suggests the use of 1900 t of PCB in open applications in Switzerland. In 1994, the BUWAL stated that this amount is probably too high for Switzerland because, for example, polymer processing in Switzerland was done on a much smaller scale than in Germany [41]. Also, Switzerland did not produce carbon-less copy paper, another known open application of PCBs. The assumed amount of PCBs in open applications in the model is 1500 t.

Also for Denmark, PCB usage data is available. The Danish Environmental Protection Agency estimated in 2006 that 200 t of PCBs have been used for sealed glazing units in Denmark (Trap et al. (2006), cited in Grontmij [53]). An extrapolation of this number to Switzerland (five million inhabitants in Denmark in 1975, 6.3 million in Switzerland) gives a usage of 250 t of PCBs in sealed glazing units. The PCB mass used in joint sealants in Switzerland was estimated to be 100–300 t [39]. The values used for Switzerland (250 t) might therefore be rather an underestimation than overestimation because joint sealants were not only used in glazing units but also in other parts of buildings.

PCBs were used also in some other use categories in Switzerland, which are not described and quantified in this inventory. The BUWAL described in one of their reports the usage of Aroclor in lubricants of aircrafts [41]. According to an investigation of the Cantonal Laboratory of Zurich, Coronado aircrafts from Swissair contained lubricants (Castrol 3 C) with 1% Aroclor. It was stated that the emissions from the aircrafts were 10 kg of Aroclor per year and aircraft. The 13 Coronado aircraft from Swissair, which were in use between 1962 and 1974 [54] might therefore have emitted 70 kg of PCBs per year. This amount is 8% of the emitted PCB amount in 1962 and 2% of the emitted amount in 1974. The emissions were not included in the inventory due to missing information on the Aroclor type.

Another field of application is the use of PCBs as plasticisers and flame retardants in paints and varnishes [55]. Products containing PCBs included concrete paints and varnishes for metals, emulsion priming and top coats for use on concrete or plasterwork. The used amounts in those paints and varnishes might not have been high, but the large surface areas of the paints and varnishes make them a potentially important emission source. PCBs were also used as flame retardants and plasticizers in plastics [41]. The BUWAL reported PCB concentrations of up to 200 ppm of PCBs in shredded cables. The used amounts of PCBs in plastics in Switzerland are, however, unknown, because most of the plastics were imported to Switzerland in products [41].

These comparisons show in general that the amounts of PCBs used in specific use categories in Switzerland are realistic. However, the emission inventory most probably still underestimates the emissions, since we were not able to quantify all PCB uses.

### Emissions

As an independent model validation, the modelled emissions to air are compared to emission estimates by Bogdal et al. [51] and Diefenbacher et al. [52]. In both studies, the authors used a combination of air measurements and modelling to estimate PCB emissions from the city of Zurich i.e. the largest city in Switzerland (approx. 400’000 inhabitants) with many typical activities involving the usage of POPs. These estimates were then extrapolated to Switzerland relative to the number of inhabitants. The modelled emissions of our study are lower than the emissions estimated by these studies (Fig 6). To reach their emission estimates, the emissions to air of this study would have to be increased by a factor of 1.65. This implies that in this emission inventory, certain emissions might be underestimated or missing (see previous Section).

Another reason for our low emission estimates could be that the successful implementation of the control strategies may have taken longer than we assumed. We also did not consider that the volatilization of PCBs in open applications is temperature dependent and that elevated temperatures lead to significantly enhanced emissions. Both points are possible explanations for the observed discrepancy between our model results and the previously reported emissions to air.

The general emissions trend (increasing emissions in Switzerland until the mid-1980s and decreasing emissions afterwards) is, however, confirmed by measurements of PCB concentrations in Swiss soils. Bogdal et al. [56] reconstructed temporal trends of PCBs in archived soil samples from six sampling sites in Switzerland, covering the period 1989 to 2014. The soil concentrations of the PCBs peaked in all sampling sites in or after 1994, which was ten years after the peak of the emissions. This is reasonable as PCB concentrations in soil decrease very slowly. Bogdal et al. [56] calculated a halving time for concentrations of PCBs in soil of approximately eight years.

We also compared our emission results to data from the BUWAL [25]. Experts from BUWAL estimated the PCB emissions from abrasion, weathering, and renovation of corrosion-protected objects in Switzerland between 1960 and 1990 at around 1 t/year. This estimation is a factor of two higher than the value calculated with our model (16 t for the entire period 1960 to 1990). Emission estimates for other processes in Switzerland are unfortunately not available, but some values are reported for Germany (Section J in S1 File).

The Swiss PCB emissions to air in 2014 calculated in this study can also be compared to PCB emissions from other countries that have been reported to the UNECE CLRTAP (Table K1 in S1 File). It is notable that only eight out of 39 member parties reported emissions in NFR category 2K (consumption of POPs and heavy metals), which shows that most of the emissions estimates reported to the UNECE CLRTAP are incomplete and most probably underestimate the real emissions. Considering only those countries that reported PCB emissions to NFR category 2K, the emissions of Switzerland per capita as calculated in this study rank in the near middle of the reported emissions.

## Conclusions

The emission inventory set up in this study includes all categories of PCB usage that are relevant for Switzerland and combines them with specific disposal categories. The results point out the consequences of the use of PCBs in open applications, which have largely been previously overlooked. For example, open applications are not mentioned in the EMEP/EEA guidebook for NFR code 2K [10]. This is probably because these applications seem negligible, as the PCB amounts used in these applications were comparatively small. However, our study shows that their contribution to contemporary emissions is crucial and should not be underestimated. We would therefore like to encourage other researchers and environmental agencies to identify and quantify the relevant PCB emission sources in their countries and use our model to calculate and report PCB emissions to air.

Additionally, we could show that PCBs are still present in applications today and that there is still a need for renovation and disposal of PCBs. In particular, PCBs in open applications are not yet completely disposed of; we calculated that 35% of the amounts are still in use in joint sealants and anti-corrosive paints.

The emissions that might occur during the renovation and disposal of the remaining PCBs should, however, be smaller than emissions in the past, due to improved renovation and combustion technologies. The application of improved air filters for example has reduced the PM 2.5 emission factors of Swiss steelworks, municipal waste incinerations and hazardous waste incinerations plants by around two orders of magnitude, and we assume a similar reduction for the PCB emissions to air from these plants. Such an emissions reduction would be desirable for plants worldwide, and we would like to encourage company owners and governments to invest in technology improvements which further reduce PCB emissions.

The current decreasing trend of PCB emissions to air is caused by the expiring usage of PCBs and improved treatment and disposal technologies, and this is a necessary and important step to reduce the environmental concentrations. However, PCBs will still persist at relevant environmental concentrations for several decades due to their accumulation potential and persistence.

Future research could improve the emission model, for example, by introducing temperature-dependent emission factors or investigating missing emissions sources. For example, Hu and Hornbuckle [14] found that PCB-11 (and other PCB congeners) were inadvertently produced during the manufacturing of paints. It would be interesting to assess whether or not emissions from ‘non-technical mixtures’ are significant for Switzerland. Future research could additionally investigate if primary emissions from unintentional formation (de novo synthesis) were important between 1930 and 1980.

## Supporting information

S1 File Supporting information S1 File. The file includes detailed information about the used parameters and additional figures and tables for the result sections.
(PDF)

S2 File Supporting information S2 File. The file contains the developed model in Excel/VBA.
(XLSM)
