# Supplementary material for: Import, use, and emissions of PCBs in Switzerland from 1930 to 2100
Source: PLoS One. 2017 Oct 5;12(10):e0183768. doi: 10.1371/journal.pone.0183768 (PMC5628794; doi:10.1371/journal.pone.0183768)
Supplement: S1 File — The file includes detailed information about the used parameters and additional figures and tables for the result sections. (PDF) [file pone.0183768.s001.pdf]

Supporting Information S1: Import, use and emissions of PCBs in Switzerland from 1930 to 2100

Juliane Glüge[1], Christine Steinlin[1,2], Simone Schalles[1], Lukas Wegmann[3], Josef Tremp[4], Knut Breivik[5,6], Konrad Hungerbühler[1], and Christian Bogdal[1]

[1] *Institute for Chemical and Bioengineering, Swiss Federal Institute of Technology, ETH Zurich, 8093 Zurich, Switzerland*
[2] *Present address: EBP Schweiz AG, CH-8032 Zürich, Switzerland*
[3] *Office for Environmental Protection and Energy of the Canton of Basel-Country, CH-4410 Liestal, Switzerland*
[4] *Industrial Chemicals Section, Federal Office for the Environment, CH-3003 Bern, Switzerland*
[5] *NILU - Norwegian Institute for Air Research, Box 100, NO-2027 Kjeller, Norway*
[6] *University of Oslo, Department of Chemistry, Box 1033, NO-0315 Oslo, Norway*

Contents

A Toxic equivalency factors (TEFs) for the dl-PCBs . . . 2
B Calculation of lifespans . . . 2
C Disposal pathways . . . 4
C.1 Transformers and large capacitors . . . 4
C.2 Small capacitors . . . 5
C.3 Anti-corrosive paints . . . 6
C.4 Joint sealants . . . 7
C.5 Sewage sludge . . . 8
D Accidental release factors . . . 8
D.1 Accidental release from usage to soil . . . 8
D.2 Accidental release from usage to fire . . . 9
E Emission factors . . . 9
E.1 Emissions from usage . . . 9
E.2 Emissions from disposal . . . 10
E.3 Emissions from treatment . . . 11
E.4 Emissions from accidental release . . . 12
F Emissions to water bodies . . . 12
F.1 Amount of produced sewage sludge . . . 12
F.2 PCB concentrations in sewage sludge . . . 13
G Parameter uncertainties for Monte Carlo simulation . . . 14
G.1 Overview . . . 14
G.2 Considerations for choosing the confidence factors and standard deviations . . . 15
H Disposed PCB amounts . . . 19
I Emitted PCB amounts . . . 20
J Comparison of Swiss emissions for single categories with emissions from Germany . . . 21
K Comparison of Swiss emissions for 2014 with emissions from other countries . . . 21

A Toxic equivalency factors (TEFs) for the dl-PCBs

Table A1: Toxic equivalency factors (TEFs) for the dl-PCBs [Van den Berg et al., 1998, 2006].

PCB congeners	WHO 1998 TEF	WHO 2005 TEF
PCB-77	0.0001	0.0001
PCB-81	0.0001	0.0003
PCB-105	0.0001	0.00003
PCB-114	0.0005	0.00003
PCB-118	0.0001	0.00003
PCB-123	0.0001	0.00003
PCB-126	0.1	0.1
PCB-156	0.0005	0.00003
PCB-157	0.0005	0.00003
PCB-167	0.00001	0.00003
PCB-169	0.01	0.03
PCB-189	0.0001	0.00003

B Calculation of lifespans

The average lifespans of the five usage categories are defined according to three sets of information: i) the reported mass of PCBs in use in a specific category and a specific year (Table B1, Fig. B1), ii) the reported mass of PCBs disposed of from a specific category in a specific year (Table B1, Fig. B1), and iii) the lifespans of the products in the usage categories reported in literature (see below).

The mass of PCBs in use in transformers was estimated to be less than 10 t in 2005 (Fig. B1) [Kohler et al., 2005]. In 1997–1999, 21 t of PCBs were disposed of from transformers per year [Tremp and Wegmann, 2001]. These values of usage and disposal are reached when the lifespan of transformers in the model is set to 25 years and a disposal term from 1993 onwards is introduced to reflect the shorter usage time due to regulatory pressure. In Switzerland, PCB-containing transformers and capacitors heavier than one kg were supposed to be put out of operation by 1998 [Bundesrat, 1986]. We modelled this by shifting 1% of the PCBs, which were still in use in transformers in 1993 to the disposal categories. The percentage was then increased by 1% per year (being 6% in 1998 and 10% in 2003). The obtained lifespan corresponds well with reported data from literature which recommend a lifespan of transformers of 25 to 30 years [Breivik et al., 2002; Bürgin, 2002; Harrad et al., 1994].

For large capacitors, the mass of PCBs in use was estimated to be between 200 and 600 t in 1999 (Fig. B1) [Tremp and Wegmann, 2001]. In contrast, another study estimated the mass of PCBs in use to be 0.7 t in 2002–2004, however, the author stated that this value might be underestimated [Wegmann, 2005]. For the period 1997–1999, a disposal of 14.3 t PCBs/year has been identified for large capacitors (Fig. B1) [Tremp and Wegmann, 2001]. To model usage and disposal in the range of these values, the lifespan of large capacitors has to be set to 25 years. This corresponds to literature values of 20–25 years [Breivik et al., 2002] and to the descriptor ‘long’ published in literature [Tremp and Wegmann, 2001; Wegmann, 2005].

The estimates for small capacitors are variable, as this usage category is diverse. The mass of PCBs in use in 1998/1999 was estimated to be 100–270 t (Fig. B1) [Kuhn and Arnet, 1998; Tremp and Wegmann, 2001]. The disposal of PCBs from fluorescent light ballasts was estimated to be 21 t PCBs/year [Kuhn and Arnet, 1998], while Eugster et al. [2007] identified a disposal of PCBs from electrical and electronic waste of 0.5–4.4 t PCBs/year. The lifespans of small capacitors depend on the product they are contained in. In household devices it was estimated to be 10–20 years [Barghoorn et al., 1988; Breivik et al., 2002; BUWAL, 1994b], while the lifespan of fluorescence lamps was estimated to be 18–30 years [Barghoorn et al., 1988; BUWAL, 1994b; Kuhn and Arnet, 1998]. Here, a lifespan of 22 years is used, which is longer than the lifespan of household devices, because fluorescence lamps are responsible for the main PCB stock in this category

[Tremp and Wegmann, 2001]. Also, when using this number, modelled usage and disposal correspond to the values from literature (Fig. B1).

For anti-corrosive paints, little information is available on the PCB mass in use in this category in specific years. Mengon and Schlatter [1993] estimated a mass of 100 t PCBs to be in use in 1999 (Fig. B1). The Swiss Federal Office for the Environment, Forests, and Landscape (BUWAL) estimated that half of the used amount was gone in 2000 [BUWAL, 2000]. This means that until present times, a high percentage of PCBs in this category is still present. This is due to the long lifespan of such paints and because steel constructions are often only partially renovated [Tremp and Wegmann, 2001]. The given amounts are achieved by modelling anti-corrosive paints with a lifespan of 45 years. A lifespan of 60 years has been estimated for structural steelwork [Davis et al., 2007]. However, the lifespan of the anti-corrosive paints on the steelworks is probably shorter due to intermediate renovations. Therefore, a lifespan of 55 years is used for anti-corrosive paints on steel constructions.

Between 1999 and 2005, 50–150 t PCBs were estimated to be in use in joint sealants (Fig. B1) [BUWAL, 2003; Kohler et al., 2005; Tremp and Wegmann, 2001]. The modelled values of usage and disposal would be in the range of the reported literature values, if we use a lifespan of 45 years for the joint sealants in buildings. However, for the category building/civil engineering, a lifespan of 60 years was estimated [Davis et al., 2007]. Based on expert judgement from several environmental consulting companies in Switzerland, we chose a lifespan of 55 years for PCBs in joint sealants.

Table B1: Reported mass of PCBs in use and disposed of from specific usage categories in specific years in Switzerland.

year	mass	category	reference
1989	max half of amount from 1986 disposed	transformers	[BUWAL, 1994b]
1999	several tons of PCBs in use	transformers	[Tremp and Wegmann, 2001]
1997–1999	disposal of 21 t PCBs/year	transformers	[Tremp and Wegmann, 2001]
2005	<10 t PCBs in use	transformers	[Kohler et al., 2005]
1989	max half of amount from 1986 disposed	large capacitors	[BUWAL, 1994b]
1999	200–600 t PCBs in use	large capacitors	[Tremp and Wegmann, 2001]
1997–1999	disposal of 14.3 t PCBs/year	large capacitors	[Tremp and Wegmann, 2001]
2002–2004	700 kg PCBs in use	large capacitors	[Wegmann, 2005]
1999	100 t PCBs in use	small capacitors	[Tremp and Wegmann, 2001], using the disposal rate of [Barghoorn et al., 1988]
1998	270 t PCBs in use (value for fluorescent light ballasts)	small capacitors	[Kuhn and Arnet, 1998]
1998–2012	disposal of 21 t PCBs/year (estimated value)	small capacitors	[Kuhn and Arnet, 1998]
2006	disposal of 500–4400 kg PCBs/year	small capacitors	[Eugster et al., 2007]
1999	<100 t PCBs in use	anti-corrosive paints	[Tremp and Wegmann, 2001]
2000	half of used amount is gone	anti-corrosive paints	[BUWAL, 2000]
1999	>50 t PCBs in use	joint sealants	[Tremp and Wegmann, 2001]
2003	100 t PCBs in use	joint sealants	[BUWAL, 2003]
2005	50–150 t PCBs in use	joint sealants	[Kohler et al., 2005]

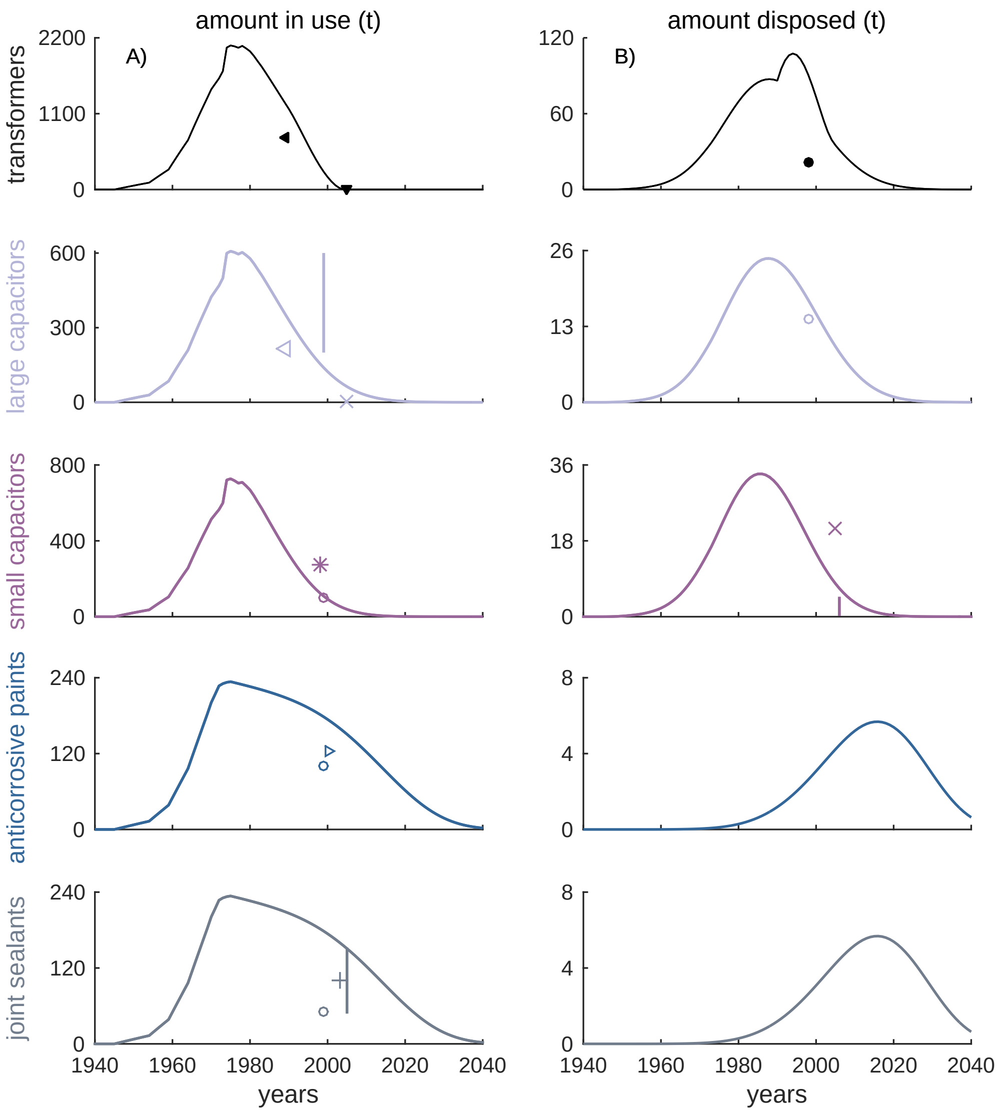

Figure B1: Left column (A): Mass of PCBs in use in the usage categories between 1940 and 2040 in the model (lines) and in the literature (dots). Right column (B): Disposed mass of PCBs from the usage categories to the treatment or disposal categories between 1940 and 2040 in the model (lines) and literature (dots). For references of the literature values please refer to Table B1.

C Disposal pathways

C.1 Transformers and large capacitors

Between 1940 and 1991, we assume transformers and large capacitors to have been partially disposed of to landfills as for example the landfill for hazardous waste in Kölliken, Aargau was only closed in 1985 (Fig. C1, Table 4, main document) [SMDK, 2016]. A part of the contaminated waste oil was also burned openly or in incineration plants that were for example used for drying grass [BUWAL, 1994b]. However, these disposal pathways have never been quantified. The percentages of landfilling, open burning, and waste incineration are set to 80/10/10 in 1940 (estimate). In 1972, the underground disposal facility Herfa Neurode in Germany opened and it is known that Switzerland exported PCBs to this disposal facility [KSEntsorgung, 2017; Tremp and Wegmann, 2001]. However, the export amounts in the early years are unknown. 57% of the PCB containing wastes were exported in 1991 [BUWAL, 1994a], so we assumed a linear increasing export, starting in 1972 and reaching 57% in 1991. The statistics of hazardous waste disposal from 1991 showed also that 43% of PCB-containing hazardous waste was treated in Switzerland. Until the mid-1990s, no facilities for the treatment of PCB-containing waste were available in Switzerland [Tremp and Wegmann, 2001]. We assumed therefore that PCB-containing hazardous waste was treated until 1997 in municipal

waste incineration plants. The percentage of PCB-containing transformers and large capacitors which has been treated between 1940 and 1991 in incinerations plants was increased linearly from 10% in 1940 to 43% in 1991. We assumed furthermore that the share of high-temperature incineration increased after 1991, while municipal waste incineration decreases, and that 100% of high-temperature incineration was reached in 2010 (Fig. C1).

Tremp and Wegmann [2001] assumed that two-third of the disposed PCB-containing transformers and capacitors were exported in 1998 and the export statistics of the BUWAL reveals that the amount of legally exported PCB-containing transformers and capacitors decreased afterwards drastically [Tremp and Wegmann, 2001]. The reason is probably that PCB-containing transformers and capacitors weighing over 1 kg had to be removed from installations and properly disposed of by mid-1998 [Bundesrat, 1986]. For the emission model we assumed a share of 67% export in 1998 (plus 27% municipal waste incinerations and 6% hazardous waste incineration) and a drop to 24% export in 1999. We assumed no legal export from 2000 on [Tremp and Wegmann, 2001] but illegal export of 21% (16% large capacitors, 34% transformer, use ration 30/70) [EEA, 2012].

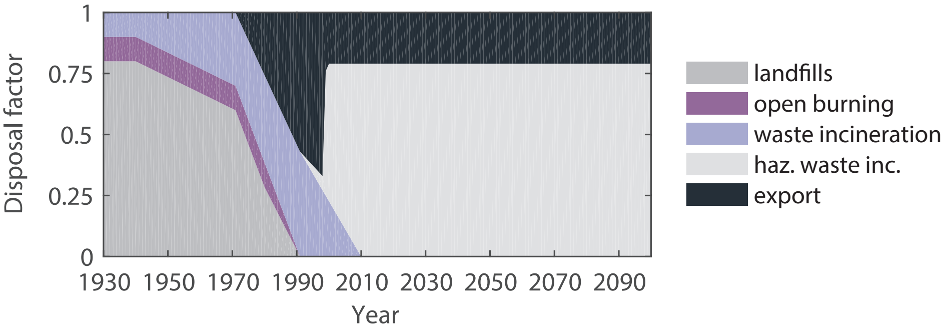

Figure C1: Disposal factors for PCBs in Switzerland for transformers and large capacitors: landfills, open burning, municipal waste incineration, export, and hazardous waste incineration.

C.2 Small capacitors

Until 1980, we assume the disposal of small capacitors to be similar to the disposal of municipal waste (Fig. C2, Table 4, main document). In 1940, we assume 20% of the municipal waste to be disposed of by open burning, 20% in municipal incinerators, and 60% on landfills. In 1969, a Swiss waste management survey revealed an allocation of communal waste to 53% incineration and 47% unregistered and registered landfills [Fahrni, 2010]. The fraction of communal waste incineration increased to 80% in 1978, while only 20% of the waste was deposited on landfills [Fahrni, 2010]. These fractions did however not consider exported PCBs. Based on the information that the underground disposal facility Herfa Neurode in Germany opened in 1972 [KSEntsorgung, 2017] and that two-third of the disposed PCB-containing transformers and capacitors were exported in 1998 (assumption from Tremp and Wegmann [2001]), we calculated that 20% of the PCB-containing small capacitors were exported in 1978 (linear interpolation). The percentages for municipal waste incineration and landfilling in 1978 were therefore 68% and 12%, respectively.

In 1998, the year of the ordinance on the return, the taking back and the disposal of electrical and electronic equipment, a study about the disposal of fluorescent light ballasts in the Canton Aargau showed that due to lack of knowledge, 83% of the ballasts were disposed of in shredder facilities, 10% were treated in special facilities, and 7% destroyed by high-temperature incineration [Kuhn and Arnet, 1998]. We assume here that the treatment in special facilities included separation of the PCB-containing parts followed by incineration as municipal waste (Fig. C2), and that the PCBs reaching the shredder facilities were afterwards contained in the shredder residue (RESH) and were either disposed of on landfills (16%) or exported (67%) (Fig. C2). Fluorescent light ballasts are taken here as representatives for small capacitors, as they were widely used, frequently contained PCBs, and are in use for a long time, making them responsible for the largest PCB mass of the small capacitors [Barghoorn et al., 1988; Tremp and Wegmann, 2001].

Since 2000, the legal prohibition of disposing combustible waste on landfills became effective, and as a

consequence, RESH was burned in municipal waste incinerators [Kuhn and Arnet, 1998]. A part was also exported but the export statistic show a drastically decreasing trend for the PCB export after 1998 [Tremp and Wegmann, 2001]. After 2000, the recycling of electronic waste improved. Electronic waste was estimated to be disposed of to 72% in recycling (hazardous waste incineration), 25% in shredder facilities, 2% by export, and 1% in municipal waste incinerators [Morf et al., 2007]. Statistics about illegal export, however, reported that between 16% and 38% of the electronic waste was exported [EEA, 2012]. We assumed an exported fraction of 16% since Switzerland is one of the countries with the highest rate of return of electronic waste (Fig. C2). The fractions for municipal waste incineration, shredder (with municipal waste incineration) and hazardous waste incineration after recycling were assumed to be 1%, 21%, and 62%, respectively.

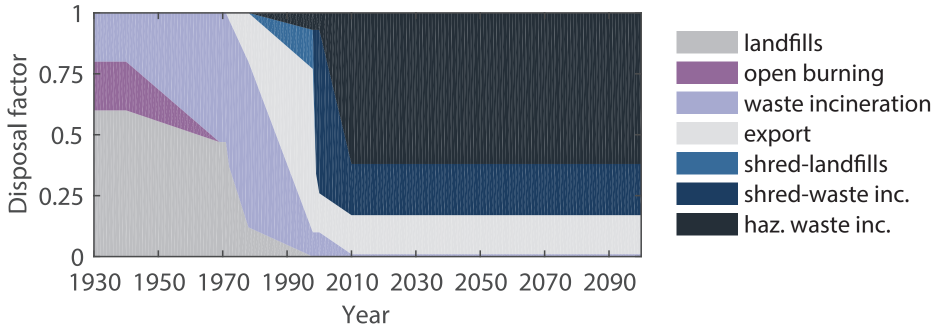

Figure C2: Disposal factors for PCBs in Switzerland for small capacitors: landfills, open burning, municipal waste incineration, export, shredder-landfills, shredder-municipal waste incineration, hazardous waste incineration.

C.3 Anti-corrosive paints

PCB-containing anti-corrosive paints were used on different steel construction including bridges, power poles, turbines, tanks and others. Some of these steel constructions are renovated (for example sandblasted) after some years and the PCBs are disposed of with the blasting media. Others, however, keep their PCB-containing anti-corrosive paints until the demolition. We assumed for the disposal that half of the PCBs, which are used in anti-corrosive paints, are disposed of during renovation and half are disposed of with the steel construction.

The BUWAL released a guideline in 1995 where they described the correct handling of anti-corrosive paints during the renovation of an object [BUWAL, 1995, 2000]. We assume therefore from 1995 on that PCB-containing paints from renovation have been treated in high-temperature incinerators (estimate) (Fig. C3, Table 4, main document). PCBs-containing paints from renovation before 1995 were assumed to be disposed of by land-filling or municipal waste incineration with increasing importance of municipal waste incineration over time (estimation: 100% disposal to landfills in 1940, 10% in 1990).
The part of the PCBs which was not removed from the object during the renovation was assumed to be disposed of with the steel constructions. Steel waste has been treated in shears (shearing) and steelworks before the beginning of the PCB production, as for example one of the largest steelworks in Switzerland was commissioned in 1918. When steel is cut into pieces, PCB paints partly peel off and remain in the residues [Hauser, 2014]. The fraction of PCBs in the residues was estimated to be 80% [Hauser, 2016]. We estimated that the residues were disposed of by landfilling or municipal waste incineration until 1995 with increasing importance of municipal waste incineration over time (estimation: 100% disposal to landfills in 1940, 10% in 1990). The PCBs on the steel fraction were assumed to be treated in the steelworks where temperatures of around 1600°C lead to the destruction of the pollutants [Hauser, 2014, 2016]. 50% of PCBs on steelworks after 1995 are assumed to have been treated in high-temperature incinerators (estimate) [BUWAL, 1995, 2000]. The other part was assumed to be disposed of to the shears. From these 50%, 20% were assumed to be treated in steelworks and 80% were assumed to be disposed of with the residues. The residues are separated in metals which are recycled, mineral parts which are deposited to landfills and organic parts which are disposed of in municipal waste incinerators [Hauser, 2014]. We assumed that 25% of the PCBs in the shear residues were disposed of with the mineral part to landfills and 75% were disposed off with the

organic parts to municipal waste incinerators.

In all years, old installations, such as for example hydraulic turbines with PCB-containing paints, were exported to developing countries. This export, or at least the PCB content of the paints, is not well documented. We estimated that 10% of the of the steel constructions were exported after 2012.

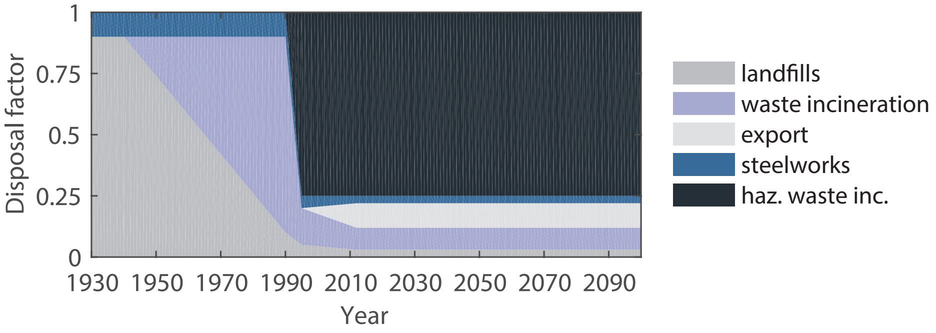

Figure C3: Disposal factors for PCBs in Switzerland for anti-corrosive paints: landfills, municipal waste incineration, export, steelworks, and hazardous waste incineration. All disposal categories first undergo the treatment step renovation (not shown here).

C.4 Joint sealants

Before 1995, joint sealants were mainly disposed of on landfills for inert matters, as joint sealants were not considered to be problematic (Fig. C4, Table 4, main document) [Tremp and Wegmann, 2001]. In this period, we estimate open burning to have been around 10% (estimate). Municipal waste incineration likely did not play an important role for joint sealants, as at that time, the PCB-containing material was not removed from the construction waste [Tremp and Wegmann, 2001]. We assume that the prohibition of disposing combustible waste on landfills in 2000 has influenced the disposal of joint sealants, leading to higher masses being disposed of in municipal waste incinerators instead of landfills. In 2003, BUWAL published a guideline about the correct disposal of PCB-containing joint sealants [BUWAL, 2003]. Therefore, we assume that since 2000, the greatest part of the PCB mass from disposed joint sealants has been disposed of correctly in hazardous waste incinerators. However, if the PCB concentration in the joint sealants is low, the waste can be disposed of in municipal waste incinerators. Due to the lack of data quantifying this amount, we estimate the fraction of PCBs disposed of in municipal waste incinerators to be 10% of the PCBs. Also, residues of PCB-containing joint sealants in construction waste disposed of on landfills can lead to an input of PCBs to landfills. This includes sealants with a low PCB concentrations or sealants that have not been tested for PCBs. Because such data is not available, we estimate this fraction to be 10% of the PCBs. All joint sealants first undergo the treatment step renovation.

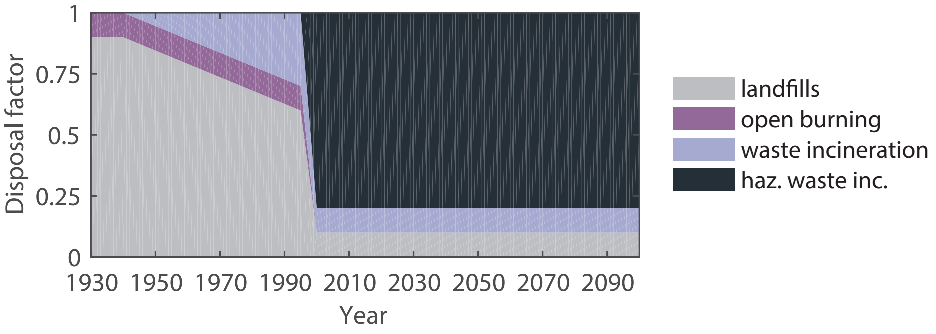

Figure C4: Disposal factors for PCBs in Switzerland for joint sealants: landfills, open burning, municipal waste incineration, and hazardous waste incineration. All disposal categories first undergo the treatment step renovation (not shown here).

C.5 Sewage sludge

The first waste water treatment plants were built in 1955, but there are no information available how the sludge was disposed of. In general there are four ways to dispose of the sewage sludge. It can be used as fertilizer in agriculture, it can be dumped to landfills, it can be incinerated in municipal or sludge incineration plants or in cement work or it can be exported. According to data by Külling et al. [2002], the share of usage in agriculture in Switzerland was 80% in 1974, 65% in 1980, 50% in 1985, 55% in 1994, and 38% in 2000 (Fig. C5). After that year a strong decrease took place and the use in agriculture dropped to 21% in 2002 [BUWAL, 2004]. Since 2006, the use of sewage sludge in agriculture in Switzerland has been banned for reasons of health and ecotoxicological risks [Kupper, 2008]. The fraction of sludge dumped on landfills was 17% in 1994 and decreased to 2% in 2000 and to 1% in 2004 due to the ban on dumping burnable waste on landfills [Morf et al., 2007]. We assume the fraction of sewage sludge on landfills to be zero after 2006. There are no data available for the period before 1994 that distinguish between the fraction dumped on landfills and incinerated. As burning the sludge in municipal, sewage incineration plants or cement works is not profitable, a decrease to a very small share back to 1980 has been estimated [Morf et al., 2007]. Therefore, the biggest part of the sludge, not used in the agriculture, was assumed to have been dumped on landfills. The amount of exported sewage sludge was estimated by [BUWAL, 2004] for 2000 and 2002 with 1% and 6%, respectively. We assumed that the export will stay at 6% also in the future.

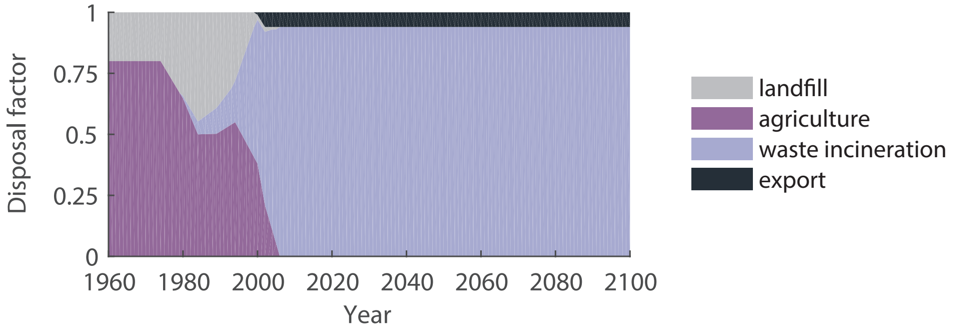

Figure C5: Disposal factors for PCBs in Switzerland for sewage sludge: landfills, agricultural fertilizer, municipal waste incineration, export.

D Accidental release factors

From each usage category, PCBs can be accidentally released. Here, this release includes two categories: soil and fire. When released to soil, PCBs are partly stored and partly degraded. In the case of fire, PCBs are partly destroyed by high temperatures and partly emitted to the environment.

D.1 Accidental release from usage to soil

The spillage rate from PCB-transformers was estimated to be 0.03%–0.06% of the total PCB mass in use in this category per year [Annema et al., 1995; Harrad et al., 1994; USEPA, 1987]. Assuming that 25% of the spillages occur to soil [Breivik et al., 2002], the accidental release factor from transformers to soil would be between 0.8e-4 and 1.5e-4 per year. The remaining 75% of the spillages occur for example inside buildings, or onto concrete followed by cleaning. The UNEP toolkit and the EMEP/EEA guidebook of 2016 report a spillage rate of 0.06 kg PCBs/t of dielectric fluid for PCB-transformers [Berdowski et al., 1997; EEA, 2016; UNEP, 2013]. Assuming a PCB content in transformer oil of 65% [Engler, 1995] and again a spillage to soil of 25%, results in an accidental release factor to soil of 2.3e-5. Breivik et al. [2002] used a factor of 0.002 per year for all closed applications (default scenario), a factor which is based on spillages from transformers and capacitors (see below). In this study, we use an accidental release factor of 1e-4 (weight/weight/year) as this lays in the range of the reported data (Table 3, main document).

For large capacitors, the spillage rate to soil was estimated to be 0.35%–1.6% of the total PCBs in use in this category per year [Annema et al., 1995; Harrad et al., 1994; USEPA, 1987]. Assuming again that 25% of the spillages occur to soil [Breivik et al., 2002], the accidental release factor from large capacitors

would be 0.001–0.004 per year. The UNEP toolkit and the EMEP/EEA guidebook recommend a spillage rate of 1.6 kg PCBs/t of dielectric fluid [EEA, 2016; UNEP, 2013]. A survey among four Swiss companies/laboratories revealed that the PCB content of capacitors varied between 20% and 30%. Using a PCB content of 25% in capacitors and again a spillage to soil of 25% gives a factor of 0.0016 per year. In this study, we use an accidental release factor of 0.002 from large capacitors to soil per year (Table 3, main document), which is the same factor as used for closed applications by Breivik et al. [2002] (default scenario). For small capacitors, no data is available for spillage to soil as this category is very heterogeneous. Annema et al. [1995] as well as Breivik et al. [2002] recommend to use the spillage rate of large capacitors for small capacitors. Therefore, we use a factor of 0.002 for accidental release from small capacitors to soil per year (Table 3, main document). Breivik et al. [2002] assumed spillage from open applications to soil to be negligible. However, other literature sources stated that the erosion from anti-corrosive paints and joint sealants by precipitation or abrasion might be important [BUWAL, 2000; Kohler et al., 2005], but this process has not been quantified. As a consequence, we use an accidental release factor of 0.002 for erosion/abrasion from anti-corrosive paints and joint sealants to soil per year.

D.2 Accidental release from usage to fire

Accidental release from usage to fire is difficult to assess, as this is a very heterogeneous and not well documented process. Breivik et al. [2002] used in the default scenario a factor of 0.001, which means that 1 out of 1000 units are destroyed by fire every year. Here, this value is used for all usage categories (Table 3, main document).

E Emission factors

E.1 Emissions from usage

Annema et al. [1995] stated that the evaporation of PCBs from a spill strongly depends on the surface onto which the chemicals are spilled to, as well as on temperature, wind speed, and the speed at which the spill is cleaned up. In a rough estimation, the authors assumed that 10% of the spilled amount is emitted to the atmosphere. Using the spillage rate of 0.06% of the total PCB mass in use in transformers discussed in the main document (Section 3.7.1), this results in an emission factor of 6.0e-5 for transformers (sum of PCBs). For capacitors, the corresponding emission factor is 1.6e-3 (spillage rate of 1.6%). Breivik et al. [2002] used these values and calculated emission factors for individual PCB congeners using congener data from Harrad et al. [1994] and the United States Environmental Protection Agency [USEPA, 1987]. The authors then used these emission factors for all closed applications, without a distinction between transformers and capacitors. In contrast, the EMEP/EEA air pollutant emission inventory guidebook of 2016 recommends no emissions to air from the usage categories transformers and capacitors [EEA, 2016]. In order to distinguish between transformers and capacitors, here, we use the emission factors of Breivik et al. [2002] divided by a factor of five for transformers, and times a factor of five for large capacitors (Table 2, main document). These numbers correspond approximately to the numbers estimated by Annema et al. [1995] for the sum of PCBs.

The emission factors for small capacitors are assumed to be the same as for large capacitors, similar to the assumption by Breivik et al. [2002]. For open applications (anti-corrosive paints and joint sealants), Breivik et al. [2002] used emission factors based on emission experiments with PCB-containing sealants. These factors are a factor of two lower than emission factor determined by Sundahl et al. [1999] for PCBs in joint sealants in a building in Sweden. The emission factor for PCB use in open applications given in Annema et al. [1995] is two orders of magnitude larger than the other values but no information is given how this factor was derived. Therefore, in this study, we use two times higher emission factors than Breivik et al. [2002] in their default scenario for PCB use in joint sealants (which corresponds to the emissions factor measured by Sundahl et al. [1999] for the sum of all PCBs). For anti-corrosive paints, we assume ten times higher emission factors than Breivik et al. [2002] in their default scenario (which corresponds to five times higher emissions factors compared to the factors for joint sealants). We assumed higher values for anti-corrosive paints due to the higher exposure of anti-corrosive paints to weathering and abrasion compared to the exposure of joint sealants in buildings.

E.2 Emissions from disposal

Emission factors for landfills are extremely difficult to quantify. On the one hand, the PCB content in a landfill is often unknown, as the material deposited is not well documented, and identifying the material by measurements is complex due to the heterogeneity of landfills. On the other hand, various types of landfills are present with different levels of protection against emissions to the environment. Also, the type of material deposited on landfills can vary tremendously. Fig. E1 summarizes emission factors for landfills reported in the literature. As expected, the values vary over several orders of magnitude. Breivik et al. [2002] calculated emission factors for a waste bed thickness of 5 m and a cap thickness of 1 m. Persson et al. [2005] measured evaporation of PCBs from the surface of a landfill only containing polysulfide sealants that have a high sorptive capacity. The authors also estimated the amount of PCBs contained in the landfill. Axelman and Broman [1999] measured evaporation of PCBs using the same flux chamber method as Persson et al. [2005] and also estimated the amount of PCBs contained in the landfill. The landfill analysed in this study contains dewatered sediments from a Bay that received paper-pulp fibres [Axelman and Broman, 1999]. The emissions were much higher than in the studies by Breivik et al. [2002] and Persson et al. [2005], a difference that was explained by the lower sorptive capacity of paperpulp fibers compared to polysulfide sealants. The study of Annema et al. [1995] reports vapour loss of Aroclor 1254 from itself and concludes that an emission of 10% of the spilled amount could be reasonable (see emissions from usage). From these 10%, we calculated emissions for individual congeners relative to the calculated emissions from a standard soil with a depth of 0.1 m (see emissions from accidental release). The values from Annema et al. [1995] would for example be valid for landfills where damaged transformers were deposited. Last but not least, Arp et al. investigated mass flows of waste by measuring PCB levels in the input of various facilities, as well as concentrations in the surrounding air. The emission factors of this study are appreciably higher than the factors from the other studies, and are not representative, because the authors quantified the emissions per newly deposited PCB mass in a year, which result in much higher emission factors [Arp, 2016; Arp et al., 2016]. For comparison, we calculated emissions from a standard soil with a depth of 5 m (see emissions from accidental release).

Here, we define a high and a low set of emission factors for landfills (black and gray stars in Fig. E1). To be in the range of the studies from the literature, we take the geometric mean of all studies except the study of Arp et al. to calculate the emission factors. For the high emission factors, we multiply the geometric mean with a factor of ten and for the low emission factors, we divide the geometric mean by a factor of ten. We use the high emission factors until the year 1980 and the low emission factors after 2050, in order to represent the improvement of landfill technologies over time. In between these years, the emission factors are linearly interpolated.

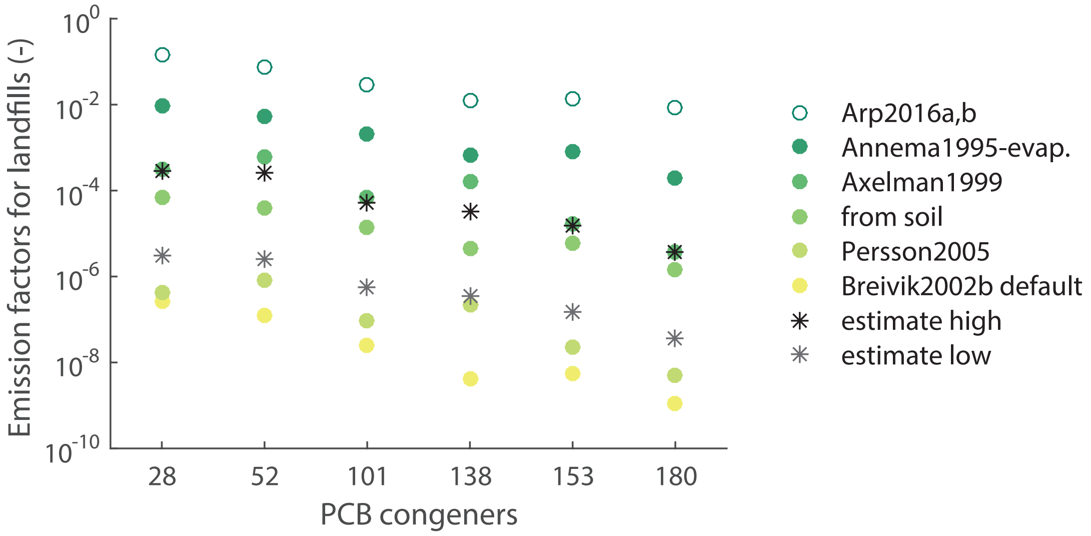

Figure E1: Emission factors for landfills as reported in the literature. The emission factors are in weight per weight per year. Note that the study of Arp et al. [2016] and Arp [2016] (open circles) calculated emission factors based on newly deposited PCB instead of PCB stock. For more information about the studies please refer to the text.

For open burning, municipal and hazardous waste incineration, emission factors in most studies are reported per mass of waste. However, the PCB content of the waste is unknown and highly variable. For this reason, we used the emission factors from the default scenario of Breivik et al. [2002] as they are reported per PCB mass instead of waste mass. Breivik et al. [2002] used data from Sakai et al. [1993] and Sakai et al. [1999] to calculate emission factors for municipal waste incineration. The emission factors for hazardous waste incineration in Breivik et al. [2002] were based on the most stringent destruction level required by hazardous waste-burning facilities in the USA. To account for technology improvements of the municipal and hazardous waste incineration plants over time, we used time variable emission factors in these two disposal categories. For this, we assumed a close correlation between PCB and PM 2.5 emission factors of the incinerations plants. PM 2.5 emission factors for some years were available from the emission information system of Switzerland (EMIS) (TableE1). For years where no data were available, data points were interpolated linearly. We used then the emission factors from Breivik et al. [2002] for PCBs for 1996 (municipal waste incineration) and 1998 (hazardous waste incineration), respectively and extrapolated the values of the other years according to the PM 2.5 emission factors.

Table E1: PM 2.5 emission factors of municipal and hazardous waste incinerations plants over time in Switzerland (source EMIS 2016)

Year/category	unit	1900	1975	1980	1984	1990	2003	2013
Municipal waste incineration	g/t	3000	500	500	450	250	9	7,2
Hazardous waste incineration	g/t	4500				250	28	28

The emission factors from steelworks were calculated from data from a Swiss steelwork. Using PCB concentrations in the exhaust air in 2014, the exhaust air volume in 2014, data about the imported steel amounts of this steelwork in 2014 and the share of this steelwork in steelworks in Switzerland in 2014, PCB emissions from steelworks for the whole of Switzerland in 2014 were around 1.87 kg. We obtained this amount for 2014 in the model by setting the emission factors from steelworks to 13 times the emission factors from municipal waste incineration (Table 2, main document). The PM 2.5 emission factors of electric arc furnaces in secondary steel production in Switzerland (Table E2) were used to calculate time variable emission factors.

Table E2: PM 2.5 emission factors of electric arc furnaces in secondary steel production in Switzerland over time (source EMIS 2016).

Year/category	unit	1900	1990	1997	1998	1999	2000	2014
Steelworks	g/t	610	610	610	22	22	6	6

The emissions of export are not included in this emission inventory, as they take place outside Switzerland.

E.3 Emissions from treatment

Emissions from shredding electric and electronic waste and vehicles have been estimated in the WAST-EFFECT project [Arp, 2016; Arp et al., 2016]. Arp et al. [2016] measured PCB levels in different waste categories and in air around waste facilities in Norway. The mass flow of PCBs was then calculated for recycling facilities, where electrical and electronic waste and vehicles were shredded and sorted. The emission factors for the six indicator PCBs in weight/weight/year were between 4.9e-3 and 8.7e-2 for the different congeners, and the emission factor for the sum of PCBs was 3.3e-2. The EMEP/EEA guidebook reports for fragmentizing operations an emission factor of 0.25 g PCBs/t of ferrous scrap recycled [EEA, 2016]. Again, this value is difficult to use, as PCB concentrations in scrap vary broadly. If we use the concentrations measured in electrical and electronic waste and vehicle fluff by Arp et al. [2016] as input, the emission factor for the sum of PCBs would be 0.05. If the concentration around 100 mg PCB/kg waste reported in Kuhn and Arnet [1998] is used, the emission factor decreases to 0.003. Here, we use the emission factors calculated

from the study of Arp et al. [2016] (Table 2, main document).

Emissions during the renovation of PCB-containing joint sealants were measured by Sundahl et al. [1999] and Åstebro et al. [2000] in buildings in Sweden. The PCBs in both studies were determined as part of projects aiming at developing clean-up methods for PCB-containing elastic sealant used in outdoor joints between concrete blocks. Sundahl et al. [1999] measured PCB indoor air concentrations, PCB concentrations in the joint sealants and PCB concentrations in the surrounding soil. They concluded from their measurements that the PCB spread to air and soil during the remedial action (with an emission protection system) was the same as the PCB spread to the indoor air during one year. The results from Åstebro et al. [2000] confirm these findings. Measurements from Sundahl et al. [1999] from situations when the emission protection system was broken (e.g. the vacuum cleaner tubing got loose) showed, however, that emissions are up to a factor of ten higher if no emission protection system was used. The use of emission protection systems for the reconstruction of joint sealants in Switzerland was requested after 2003 [BUWAL, 2003]. We used, therefore, in this study ten times higher emission factors for the renovation of joint sealants than for the use of PCBs in joint sealants between 1930 and 1999 and the emission factors for the use of PCBs in joint sealants from 2007 onwards. The values for the time period 2000 to 2006 were interpolated linearly (Table 2, main document).

Measurements of emissions from the renovation of anti-corrosive paints are not available. However, marine sediments below a bridge in Bergen, Norway which was sandblasted in the 1980, were heavily contaminated with PCBs, both in surface sediment and in deeper parts down to about 40 cm [Jartun et al., 2009]. We conclude from this that also the renovation of anti-corrosive paints without emission protection systems can lead to high emissions to air and soil. We even think that the emissions from sandblasting without emission protection systems exceed those from the renovation of joint sealants due to the higher surface area of the peeled paints compared to the joint sealants. The use of emission protection systems for the reconstruction of anti-corrosive paints in Switzerland was requested after 2002 [BUWAL, 2002]. We used, therefore, in this study ten times higher emission factors for the renovation of anti-corrosive paints than for the use of PCBs in anti-corrosive paints between 1930 and 1998 and the emission factors for the use of PCBs in anti-corrosive paints from 2006 onwards. The values for the time period 1999 to 2005 were interpolated linearly (Table 2, main document).

E.4 Emissions from accidental release

Emissions from soil were calculated according to Mackay and Paterson [1991] using a standard soil with a depth of 10 cm, a density of 1500 kg/m^3, and an air fraction of 20% (Table 2, main document). Sorption to soil was described as a function of the organic carbon-water partition coefficient, which is derived from the octanol-water partition coefficient [Schwarzenbach et al., 2003]. Diffusion is assumed to occur in the pore air. The resulting emission factors are between 7.0e-5 and 3.4e-3 for the different congeners. Emission factors from fire are set equal to the emission factors from open burning. This assumption has previously been used by Breivik et al. [2002].

F Emissions to water bodies

Release to water bodies is only partly included in the model. We omitted emissions to natural water bodies and quantified only the amount of PCBs contained in sewage sludge. The amount of PCBs in sewage sludge depends on two factors. The amount of produced sewage sludge and the PCB concentration in the sludge. The following two subsections describe both factors and the data used.

F.1 Amount of produced sewage sludge

The first water protection law came in force in Switzerland in 1955 with the defined goals that water in all rivers and lakes should have a sufficient quality for fishes, for the production of drinking water and for irrigation in agriculture [Fahrni, 2011]. However, the construction of treatment plants was rather slow in the

first years and we estimate that the amount of produced sludge increased to only 10 000 t until 1971. The federal parliament enacted in 1971 a new water protection act with the goal to encourage and to accelerate the construction of the necessary treatment plants. This policy was successful and about 900 treatment plants were built [Fahrni, 2011]. The sludge production increased to 90 000 t in 1974, 170 000 t in 1980, 176 000 t in 1984. and 213 000 t in 1989 [Külling et al., 2002]. The production amounts stayed then on this level and the available data indicate only small variation in between the years (211 000 t in 1994, 209 000 t in 1999 [Külling et al., 2002], 202 757 t in 2000, 199 503 t in 2002, 204 000 t in 2004 [BUWAL, 2004] and 210 000 t in 2011 [Fahrni, 2011]). We assume that the production amounts after 2011 were on the same level as in 2011.

F.2 PCB concentrations in sewage sludge

PCB concentrations in sewage sludge in Switzerland are available from Berset and Holzer [1995, 1996, 1999]; Marcomini et al. [1989], and Zennegg et al. [2013] (Fig. F1). However, the data of Berset and Holzer [1996] were reported in ng/kg dw instead of ng/g dw as shown in Fig. F1. We assumed them to be ng/g dw, because it is very unlikely that the concentrations were three orders of magnitude lower than all other reported concentrations in sewage sludge in Switzerland. The measurements from Berset and Holzer [1995]; Marcomini et al. [1989] and Berset and Holzer [1999] seemed to be fine, but since they have been done 20 years ago, we decided to use only the most recent study of Zennegg et al. [2013]. The data of Zennegg et al. [2013] cover the time period 1993 to 2012 and encompass in total 32 samples from Swiss waste water treatment plants.

PCBs in sewage sludge in Switzerland are expected to originate mainly from the use of anti-corrosive paints, but also from the use of joint sealants, the use of recycled toilet paper, and from landfill leachate. Based on this considerations, we expect PCB concentrations in sewage sludge to depend at least partly on the amount of PCBs in use in anti-corrosive paints. Fig. F2 shows the measured iPCB concentrations from Zennegg et al. [2013] in sewage sludge as a function of the estimated amount of PCBs in use in anti-corrosive paints from the same year. The data are too scattered to fit a statistical model, but they give an indication what concentrations should be expected if only the amount of PCB in use in anti-corrosive paints is known for a certain year. We set the intercept to zero to make this approach more robust.

To use the whole approach in the emission inventory model, we set up the correlation separately for every PCB congener, for which data were available. The logarithmic slopes as a function of the number of chlorine atoms of the congener are shown in Fig F3. Slopes were available for the six iPCB congeners and the 12 dl-PCB congeners. The averages for PCB congeners with 3, 4, 5, 6, and 7 chlorine atoms were 4.66, 2.39, 1.86, 0.47, and 0.14, respectively. We used the averages for PCB congeners for which no data were available and the original slopes for the iPCB and dl-PCB congeners.

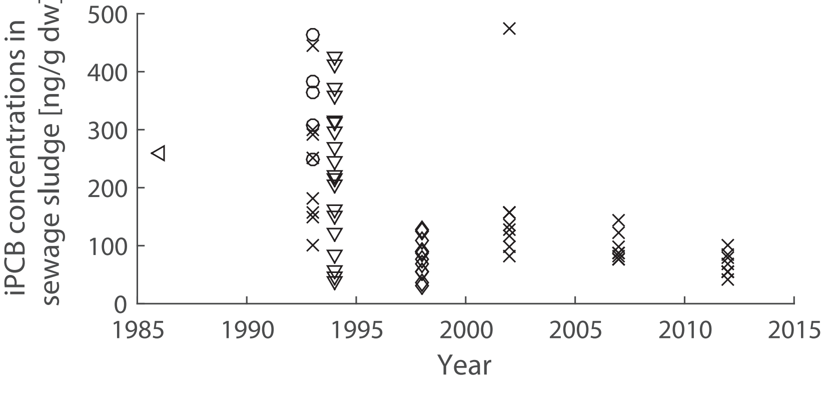

Figure F1: iPCB concentrations in Swiss sewage sludge as a function of time. o Berset and Holzer [1995], ▽ Berset and Holzer [1996], ◇ Berset and Holzer [1999], ◁ Marcomini et al. [1989], x Zennegg et al. [2013]. Concentrations reported in Berset and Holzer [1996] were assumed to be ng/g dw instead of ng/kg dw.

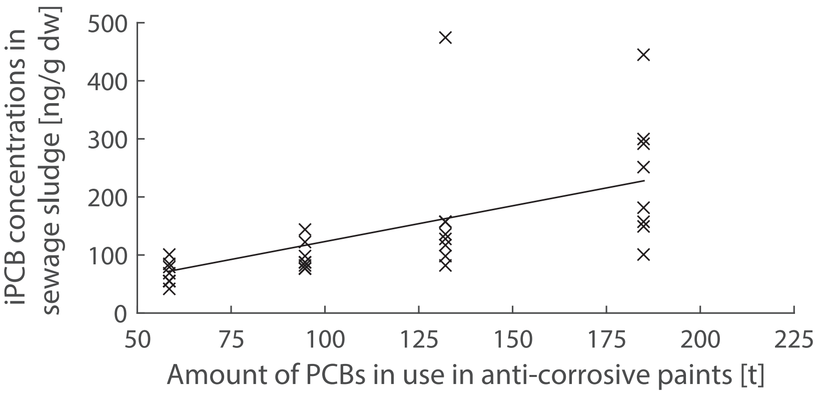

Figure F2: iPCB concentrations in Swiss sewage sludge as a function of the amount of PCBs in use in anti-corrosive paints in Switzerland in the year of the sludge concentration measurement. Data from Zennegg et al. [2013].

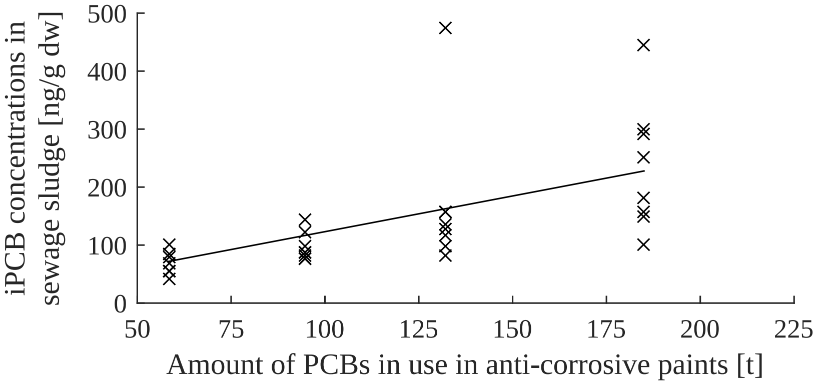

Figure F3: Logarithmic slopes between the PCB concentrations in sewage sludge and the amount of the PCBs in use in paints as function of the number of chlorine atoms in the specific PCB congeners.

G Parameter uncertainties for Monte Carlo simulation

G.1 Overview

Uncertainties in the model were calculated using a Monte Carlo simulation. For this, we defined for all parameters a distribution function and an uncertainty factor. This factor was the confidence factor (Cf) [MacLeod et al., 2002] for log-normally distributed parameters or the standard deviation (σ) for normally distributed parameters (Table G1).The simulations where performed using Latin hypercube sampling and a sampling size of 250 [Helton and Davis, 2003]. The Latin hypercube sampling was programmed in Microsoft Visual Basic for Applications using the Worksheet Function $Norm\_Inv$. This function returns the inverse of the normal cumulative distribution for a specified mean and standard deviation. It requires as third argument a probability corresponding to the normal distribution.

The 250 samples for parameters with an assumed normal distribution were generated using the value of the parameter as mean and the standard deviation of this parameter from Table G1.
$x = WorksheetFunction.Norm_Inv(Rnd(), \mu, \sigma)$
The probability corresponding to the normal distribution was generated by the function $Rnd()$ which returns a random number between zero and one. The 250 samples for parameters with an assumed log-normal distribution were generated using the logarithmic value of the parameter as mean and the logarithmic confidence factor (Table G1) times 0.5 as standard deviation. Again, the probability corresponding to the normal distribution was generated by the function $Rnd()$. The returned value from the function $Norm\_Inv$ was afterwards back-transformed using the function exp.

Table G1: Parameters in the model with their assumed distribution function and confidence factor (Cf) or standard deviation (σ)

Parameter	distribution	Cf	σ
fractions of PCB congener groups in a specific use category	normal		1.5
imported amount	log-normal	1.5	
usage factors	normal		0.01
average lifespans of the five usage categories	normal		1.1
disposal factors	normal		0.01
accidental release factor from usage to soil	log-normal	10	
accidental release factor from usage to fire	log-normal	10	
degradation in soil and landfill	log-normal	5	
emission factors for PCB use in transformers	log-normal	20	
emission factors for PCB use in large capacitors	log-normal	20	
emission factors for PCB use in small capacitors	log-normal	20	
emission factors for PCB use in anti-corrosive paints	log-normal	5	
emission factors for PCB use in joint sealants	log-normal	3	
emission factors for shredding	log-normal	3	
emission factors for renovation (before disposal)	log-normal	5	
emission factors for landfill	log-normal	20	
emission factors for open burning	log-normal	10	
emission factors for municipal waste incineration	log-normal	5	
emission factors for hazardous waste incineration	log-normal	5	
emission factors for steelworks	log-normal	3	
emission factors for soil after accidental release	log-normal	2	
emission factors for fire after accidental release	log-normal	10	
amount of produced sewage sludge	log-normal	1.3	
slopes to calculate sewage sludge concentrations	log-normal	2.6	

G.2 Considerations for choosing the confidence factors and standard deviations

If the data are normally distributed, about 95 percent are within two standard deviations. If the data are log-normally distributed, 95% of all values in the distribution lie between the median divided by the confidence factor and the median times the confidence factor [MacLeod et al., 2002].

Fractions of PCB congener groups in a specific use category (for the emissions of all 209 congeners) The fraction of a congener group (e.g. PCBs with six chlorine atoms) in a specific use category was calculated by assuming that certain technical mixtures were used in this use categories. This assumption is, however, a bit vague because different manufacturer of e.g. joint sealants might have used different PCB mixtures. However, the function of the PCBs in this use category and thus the required properties (e.g. degree of chlorination) should have been similar. The fractions are given in percentage and vary between 0% and 32% with a mean of 16.7%. They add up to 100% for every year. We assumed for the Monte Carlo simulations that 95% of all values were in between plus/minus three (standard deviation of 1.5).

Imported amount The BUWAL stated in 2000 that 6000 t of PCBs were used in Switzerland between 1930 and 1980. They assumed that 4000 of the 6000 t have been used in closed applications (transformers, large capacitors) and around 2000 t in open applications (small capacitors, plasticisers, paints, joint sealants, flame retardants, etc.) [BUWAL, 2000]. Numbers from other sources [BUWAL, 1988, 2000, 2003] for individual use categories do, however, only sum up to 5000 t of PCBs. The uncertainties of the amounts in the specific use categories are, however, a bit larger. We used therefore a log-normal distribution and a confidence factor of 1.5 to account for this differences.

Usage factors The usage factor of a specific year and use category is the fraction of the used PCB amount in this category in relation to the total amount of PCBs used in this year. The values are quite well defined because the total amounts of used PCBs in the different use categories are known. The values for the five use categories vary between 0.07 and 0.6 and add up to 1 for every year. We assumed for the Monte Carlo simulations that 95% of all values were in between plus/minus 0.02 (standard deviation of 0.01). This uncertainty probably underestimates the uncertainty of the large usage factors (>0.4) and overestimates the uncertainty of the small usage factors (<0.4). However, it was not possible to use a different approach, because the usage factors from the five use categories have to sum up to one, and the 250 values for each use factor have to correspond to a normal distribution.

Average lifespans of the five usage categories The average lifespans of the five usage categories were defined according to three sets of information: i) the reported mass of PCBs in use in a specific category and a specific year, ii) the reported mass of PCBs disposed of from a specific category in a specific year, and iii) the reported lifespans of the products in the usage categories from literature. It is difficult to define how uncertain the resulting values are – we assumed a log-normal distribution and a confidence factor of 1.1 in the Monte Carlo simulation. This means e.g. for anti-corrosive paints on steel constructions with an average lifespans of 45 years that 95% of the average lifespans in the distribution were between 41 and 50 years. These values, however, must not be mixed with the distribution of the paints lifespan. (95% of the paints on steel constructions are in use between 24 years and 56 years, if the average lifespan is 45 years).

Disposal factors The disposal factor for a specific year, disposal category, and use category is the fraction of the disposed PCB amount in this disposal and use category in relation to the total amount of PCBs disposed of in this year and use category. We tried to define these values as good as possible and included information from legislations as well as from waste disposal studies. However, we do not know how accurate the resulting values are. We assumed for the Monte Carlo simulations that 95% of all values were in between plus/minus 0.02 (standard deviation of 0.01). (The values for the different disposal categories vary between 0.01 and 0.9. and add up to 1 for every year and use category.) This uncertainty probably underestimates the uncertainty of the large disposal factors (>0.5) and overestimates the uncertainty of the small disposal factors (<0.5) However, similar to the usage factors, it was also here not possible to use a different approach, because the disposal factors have to sum up to one, and the 250 values for each disposal factor have to correspond to a normal distribution.

Accidental release factor from usage to soil The different studies for spillage from PCB-containing transformers (Section E.1) report spillage rates between 2.3e-5 and 2e-3 [Annema et al., 1995; Berdowski et al., 1997; Breivik et al., 2002; EEA, 2016; Harrad et al., 1994; UNEP, 2013; USEPA, 1987]. The value used in this study is 1e-4. Studies for spillage from PCB-containing large capacitors report spillage rates between 2.5e-4 [Annema et al., 1995] and 4e-3 [Annema et al., 1995; Harrad et al., 1994; USEPA, 1987]. The value used in this study is 2e-3. We assumed therefore that a log-normal distribution and a confidence factor of ten are appropriate for spillage from closed applications to soil. No data is available for spillage from small capacitors to soil. Annema et al. [1995] as well as Breivik et al. [2002] recommend to use the spillage rate of large capacitors for small capacitors. We decided to use also the uncertainty range of the spillage rate of large capacitors for small capacitors although this might under or over predict the uncertainties. The spillage from open applications to soil (due to erosion/ abrasion) was estimated as 2e-3. Again no data are available to verify this number, so we assumed again a log-normal distribution and a confidence factor of ten.

Accidental release factor from usage to fire Accidental release from usage to fire is difficult to assess, as this is a very heterogeneous and not well documented process. Breivik et al. [2002] used in the default scenario a factor of 0.001 which we used also for our study. We assumed in the absence of data a log-normal distribution of the values with a confidence factor of ten.

Degradation in soil and landfill Degradation rates of persistent chemicals are difficult to determine. Additionally, they depend on factors like temperature or moisture. We think, however, that the order

of magnitude of the degradation rates are correct and chose therefore a log-normal distribution with a confidence factor of five for the Monte Carlo simulation.

Emission factors for PCB use in transformers The emission factors for PCB use in transformers were estimated using a spillage rate of 0.06% [Annema et al., 1995] and the rough estimation that 10% of the spilled amount is emitted to the atmosphere. The spillage rates were assumed to be log-normally distributed with a confidence factor of ten. Adding the uncertainties for the evaporation rate, we assume the emission factors for use in transformers to be log-normally distributed with a confidence factor of 20.

Emission factors for PCB use in small and large capacitors The emission factors for PCB use in small and large capacitors are based on the same considerations as the emission factors for PCB use in transformers. We assumed therefore also for the emission factors for PCB use in small and large capacitors a log-normal distribution and a confidence factor of 20.

Emission factors for PCB use in joint sealants The emission factors for the PCB use in joint sealants are based on emission experiments with PCB-containing sealants. There were additionally validated with measurements from a building in Sweden which contained PCBs in joint sealants [Sundahl et al., 1999]. We assume therefore a log-normal distribution with a confidence factor of three for the Monte Carlo simulation.

Emission factors for PCB use in anti-corrosive paints Emission factors for the PCB use in anti-corrosive paints are based on the emission factors for PCB use in joint sealants. The emission factors were enhanced by a factor of five to account for higher emission due to higher abrasion and weathering. We assume therefore a log-normal distribution with a confidence factor of five for the Monte Carlo simulation.

Emission factors for shredding Emission factors for disposal of small capacitors to shredders were determined in the WASTEEFFECT project of Arp et al. [Arp, 2016; Arp et al., 2016]. We assume these values to be relative well defined and assume therefore a log-normal distribution with a confidence factor of three for the Monte Carlo simulation.

Emission factors for renovation of anti-corrosive paints and joint sealants PCB emissions during the renovation of joint sealants were measured in two Swedish studies [Åstebro et al., 2000; Sundahl et al., 1999], but there is, to our knowledge, no study available which reports PCB emissions during the renovation of anti-corrosive paints. The two studies on the renovation of joint sealant show quite similar results (factor of two difference), but only the study of [Sundahl et al., 1999] measured emissions during the remedial work without emission protection system. We assume therefore that the emissions factors for the renovation of anti-corrosive paints and joint sealants are log-normally distributed with a confidence factor of five.

Emission factors for landfills Emission factors for landfills are extremely difficult to quantify. Various types of landfills are present with different levels of protection against emissions to the environment. Also, the type of material deposited on landfills can vary tremendously. The emission factors in this study are the geometric mean of all applicable studies times a factor of ten (1930 to 1980) and divided by a factor of ten (2050 to 2100). Years in-between were interpolated linearly. We assumed a log-normal distribution for the Monte Carlo analysis and calculated from the range of the applicable studies a confidence factor of 20 for the emission factors for landfills.

Emission factors for open burning For open burning, the emission factors derived in [Breivik et al., 2002] were used. It is unclear how reliable these emission factors are, so we assumed a log-normal distribution with a confidence factor of ten for the Monte Carlo simulations.

Emission factors for municipal waste incineration The emission factors for municipal waste incineration are based on measurements from Sakai et al. [1993] and Sakai et al. [1999]. To account for technology improvements of the municipal waste incineration plants over time, time variable emission factors were used,

based on the PM 2.5 concentration in the exhaust air. We think that the published emission factors are reliable values for the incineration plants where they were determined for. However, they are based on measurements from only two municipal incineration plants, which decreases their applicability to general models. We assumed therefore a log-normal distribution for the Monte Carlo analysis with a confidence factor of five.

Emission factors for hazardous waste incineration The emission factors for hazardous waste incineration were based on the most stringent destruction level required by hazardous waste-burning facilities in the USA. It is unknown how close Swiss hazardous waste incineration plants are to these destruction levels, so we assumed a log-normal distribution for the Monte Carlo analysis with a confidence factor of five for the emission factors for hazardous waste incineration.

Emission factors for steelworks The emission factors from steelworks were calculated from emission data from a Swiss steelwork and the modelled amount of disposed PCBs to the steelworks in Switzerland. The PM 2.5 emission factors were used to calculate time variable emission factors. We think that the emission factors for steelworks are quite well defined although the disposed amount of PCBs originates from the inventory model and the emitted amount is based on measurements. We assume therefore a log-normal distribution with a confidence factor of three for the Monte Carlo simulation.

Emission factors for soil after accidental release Emission from soil were calculated using a modelling approach which takes into account soil depth, soil density and the air faction in soil. Not considered are the soil type, if the soil is covered or not and the temperature. We assume there fore a log-normal distribution with a confidence factor of two five in the Monte Carlo simulation.

Emission factors for fire after accidental release Emission factors from fire were set equal to the emission factors from open burning. We assumed therefore the same distribution function (log-normal) and confidence factor (ten) in the Monte Carlo simulation as for open burning.

Amount of produced sewage sludge The amount of produced sewage sludge for the time period 1974 to 2011 was taken from BUWAL [2004]; Fahrni [2011], and Külling et al. [2002]. Data for the years 1994 to 2011 from different literature sources were all in same range with variations of less than 6%. However, we assume that reported amounts from the past are not as accurate as the more recent numbers. We used therefore an log-normal distribution with a confidence of 1.3 in the Monte Carlo simulation.

Slopes to calculate sewage sludge concentrations The slope between the PCB concentration in sewage sludge and the amount of PCBs in use in anti-corrosive paints was calculated for 18 PCB congeners and grouped according to the number of chlorine atoms of the PCB congener. We obtained a log-normal distribution with confidence factors of 3.2, 4.3, 1.7, and 1.0 for PCB congeners with 4, 5, 6, and 7 chlorine atoms, respectively. The average of the four confidence factors (2.6) was used in the Monte Carlo simulation.

H Disposed PCB amounts

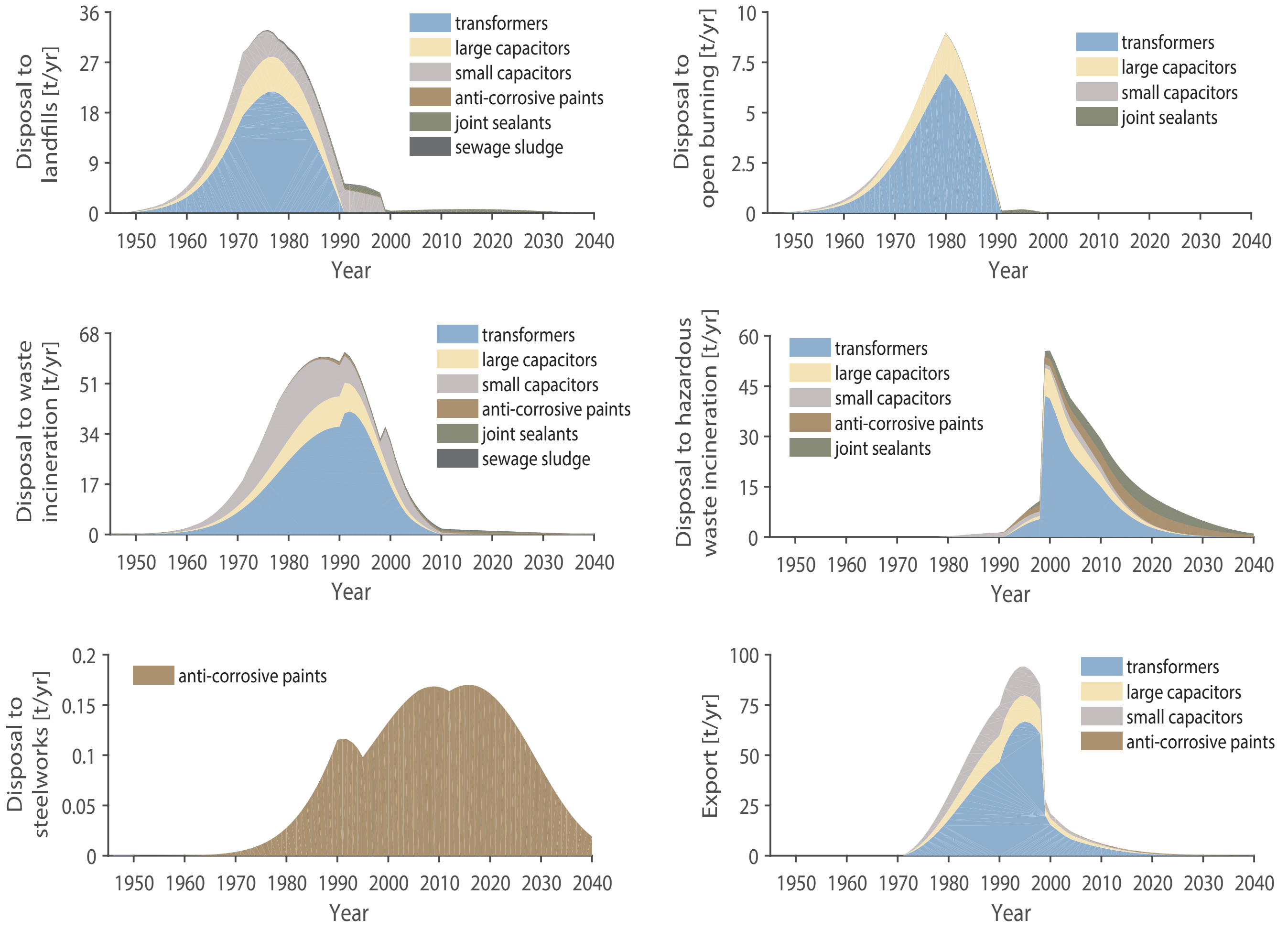

Figure H1: Masses of PCBs disposed of to the six disposal categories landfills, open burning, municipal waste incineration, hazardous waste incineration, steelworks, and export. The legend shows the origin of the PCB masses.

I Emitted PCB amounts

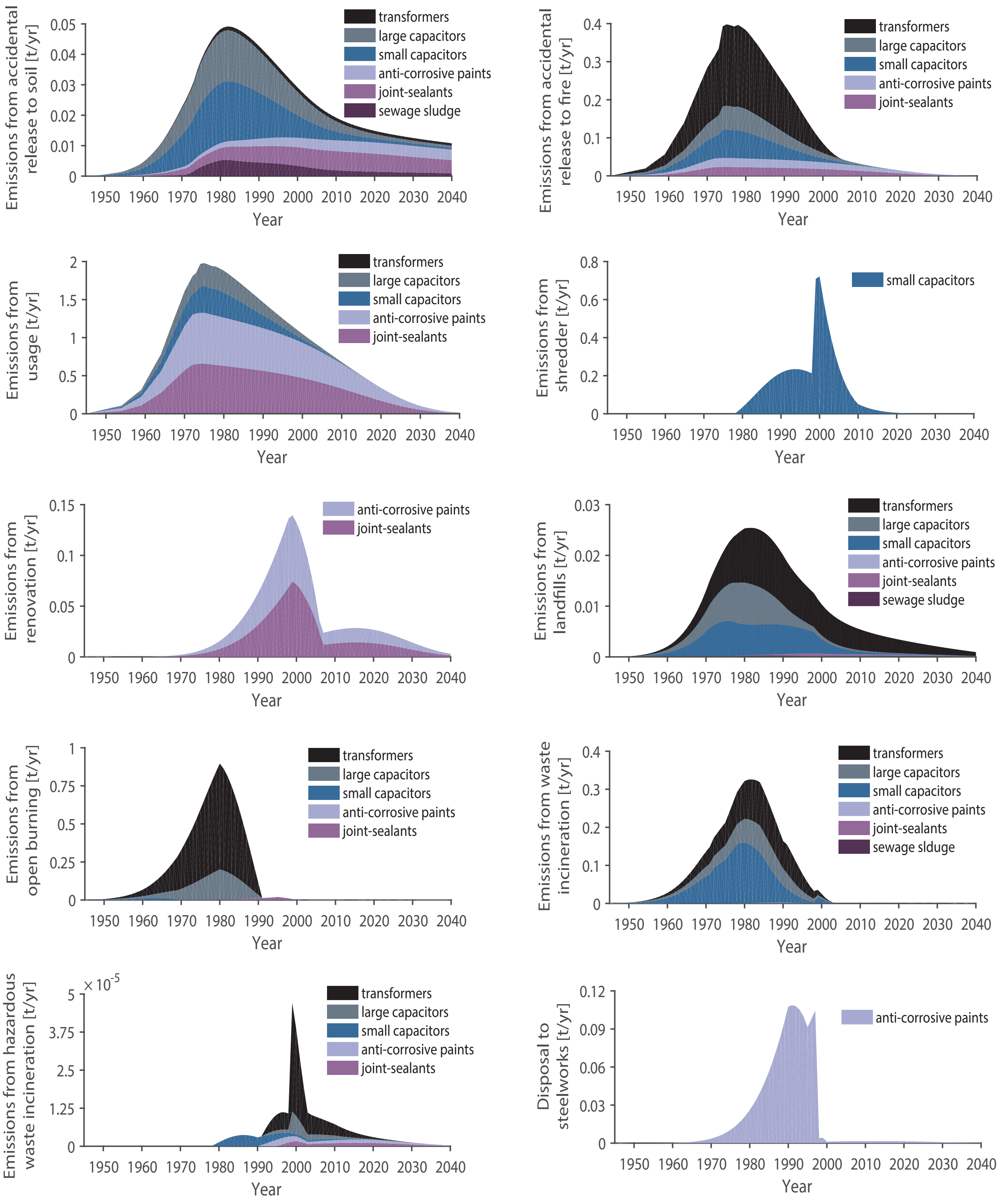

Figure I1: Emissions of PCBs from the life stages usage, accidental release to soil, accidental release to fire, disposal to landfills, to open burning, to municipal waste incineration, to hazardous waste incineration, to steelworks, and renovation and shredding. The legend shows the origin of the PCB emitted.

J Comparison of Swiss emissions for single categories with emissions from Germany

The German Federal Office for the Environment (German UBA) estimated for Germany that 5.8 kg of PCBs were released from landfilled waste in 1994 (39.8 million tons municipal waste, 93.2 m^3 landfill gas/t waste and 1.6 $\mu g/m^3$ landfill gas) [Ifeu, 1998]. Extrapolating this number to Switzerland (81 million inhabitants in Germany in 1994, 7 million in Switzerland in 1994) gives 0.5 kg of PCBs emissions from landfills. The modelled PCB emissions from landfills for Switzerland for 1994 are 15.7 kg of PCBs and are thus around one order of magnitude higher. The German UBA estimated also the emissions from municipal waste incineration in 1994. They assumed that 12 out of the 40 incineration plants satisfied the threshold limits for chlorinated dioxins and assumed for these plants emissions of 3.3 ng PCB/m^3 air. They assumed furthermore that the other incineration plants emitted 10 ng PCB/m^3 air. Based on an exhaust air volume of 1 billion m^3 per year and incineration plant, they calculated emissions of 320 g of PCBs for 1994 [Ifeu, 1998]. An extrapolation (with the number of inhabitants) of this number to Switzerland gives 27.5 kg of PCBs emissions from incineration plants. The modelled PCB emissions from municipal waste incineration in Switzerland for 1994 are 81.7 kg of PCBs and are thus a factor of three higher than the extrapolated emissions from Germany. Regarding PCB emission from hazardous waste disposal, the German UBA stated that they assume PCB emissions from hazardous waste disposal to be not important [Ifeu, 1998]. They reasoned this with clearly lower dioxin emissions from hazardous waste incineration compared to municipal waste incineration and a correlation between dioxin and PCB emissions. The modelled PCB emissions from hazardous waste incineration in Switzerland for 1994 are 0.01 kg of PCBs and are therefore three orders of magnitude lower than the emissions from municipal waste incineration for 1994. This verifies the assumption of the German UBA that PCB emissions from hazardous waste disposal are negligible compared to other emission sources.

K Comparison of Swiss emissions for 2014 with emissions from other countries

The Swiss PCB emissions in 2014 calculated in this study were compared to PCB emissions from other countries that have been reported to the Convention on Long-range Transboundary Air Pollution (Table K1). We show here the national total PCB emissions for 2014 from all source categories reported in the WebDab database [WebDab, 2016].

Table K1: Submitted PCB emissions for 2014 (national total) and PCB emissions of Switzerland calculated in this study. Countries in gray reported PCB emissions in NFR category 2K. The population numbers were taken from the World Bank Open Data [WorldBank, 2016].

Country	PCB emissions [kg/year]	population [millions]	PCB emission per capita [kg/million capita/year]
Armenia	12.24	3.017	4.06
Austria	218.3	8.611	25.4
Azerbaijan	3.02	9.651	0.31
Belgium	11.04	11.29	0.98
Bulgaria	3.39	7.178	0.47
Canada	NR	35.85	NR
Croatia	428.6	4.224	102
Cyprus	0.05	1.165	0.04
Czech Republic	3.474	10.55	0.33
Denmark	44.34	5.676	7.80
Estonia	4.223	1.312	3.22
Finland	154.5	5.482	28.2
France	48.19	66.81	0.72
Georgia	4.497	3.679	1.22
Germany	236.0	81.41	3.23
Hungary	8.823	9.845	0.90
Iceland	NE	0.33	NE
Ireland	15.987	4.64	3.44

Italy	198.1	60.80	3.26
Kazakhstan in the former official EMEP domain	151.2	17.54	8.62
Kyrgyzstan	NA	5.957	NA
Latvia	0.257	1.978	0.13
Lithuania	296.7	2.910	102
Luxembourg	2.37	0.570	4.16
Malta	0.0002	0.431	0.00
Netherlands	0	16.94	0
Norway	26.06	5.196	5.02
Poland	685.2	38.00	18.0
Portugal	410.9	10.35	39.7
Republic of Moldova	406.5	3.554	114
Romania	28.91	19.83	1.46
Serbia	724.5	7.098	102
Slovakia	58.48	5.424	10.8
Slovenia	147.6	2.064	71.5
Spain	26.85	46.42	0.58
Sweden	9.04	9.799	0.92
Switzerland (reported)	NE	8.287	NE
Switzerland (this study)	642	8.287	77.5
The former Yugoslav Republic of Macedonia	3.219	2.078	1.55
Ukraine	0	45.20	0
United Kingdom	732.5	65.14	11.2

References

Annema, J. A., Baart, A. C., Bakker, D. J., Beurskens, J. E. M., Berdowski, J. J. M., Bodar, C., van Duijvenbooden, W., Klein, A. E., Liem, A. K. D., van der Linden, A. M. A., Noordijk, H., and van de Poel, P. Evaluation of PCB fluxes in the environment. Technical report, National Institute of Public Health and Environmental Protection, Bilthoven, Netherlands, Bilthoven, Netherlands, 1995. URL http://www.rivm.nl/dsresource?objectid=rivmp:188862&type=org&disposition=inline&ns_nc=1.

Arp, H. P. Personal communication, June 2016, 2016.

Arp, H. P., Morin, N., Okkenhaug, G., Hale, S., Breedveld, G., Sparrevik, M., Almås, Å., Andersson, P., Wania, F., and Breivik, K. WASTEFFECT – Life Cycle Effects of Emerging Contaminants in Waste – Final Report. Technical report, Oslo, Norway, 2016.

Åstebro, A., Jansson, B., and Bergstrom, U. Emissions During Replacement of PCB Containing Sealants- A Case Study. *Organohalogen Compd.*, 46:248–251, 2000.

Axelman, J. and Broman, D. Inventories and fluxes of polychlorinated biphenyls from a contaminated industrial site. *Environ. Toxicol. Chem.*, 18:1871–1881, 1999.

Barghoorn, M., Bauer, R. K., and Gössele, P. Abschlussbericht zum Forschungsvorhaben PCB-Kleinkondensatoren. Arbeitsgruppe Umweltstatistik-ARGUS an der Technischen Universität Berlin. Technical report, Berlin, Germany, 1988.

Berdowski, J. J. M., Baas, J., Bloos, J. J., Visschedijk, A. J. H., and Zandveld, P. Y. J. The European Atmospheric Emission Inventory of Heavy Metals and Persistent Organic Pollutants for 1990. Forschungsbericht 104 02 672/03. Umweltforschungsplan des Bundesministers für Umwelt, Naturschutz und Reaktorsicherheit. Technical report, Apeldoorn, Netherlands, 1997.

Berset, J. D. and Holzer, R. Organic Micropollutants in Swiss Agriculture: Distribution of Polynuclear Aromatic Hydrocarbons (PAH) and Polychlorinated Biphenyls (PCB) in Soil, Liquid Manure, Sewage Sludge and Compost Samples; a Comparative Study. *Int. J. Environ. Anal. Chem.*, 59(2-4):145–165, 1995.

Berset, J. D. and Holzer, R. Determination of coplanar and ortho substituted PCBs in some sewage sludges of Switzerland using HRGC/ECD and HRGC/MSD. *Chemosphere*, 32(12):2317–2333, 1996.

Berset, J. D. and Holzer, R. Quantitative determination of polycyclic aromatic hydrocarbons, polychlorinated biphenyls and organochlorine pesticides in sewage sludges using supercritical fluid extraction and mass spectrometric detection. *J. Chromatogr. A*, 852:545–558, 1999.

Breivik, K., Sweetman, A. J., Pacyna, J. M., and Jones, K. C. Towards a global historical emission inventory for selected PCB congeners - A mass balance approach 2. Emissions. *Sci. Total Environ.*, 290(1-3):199–224, 2002.

Bundesrat. Verordnung über umweltgefährdende Stoffe (Stoffverordnung, StoV), 1986. URL https://www.admin.ch/opc/de/classified-compilation/19860123/200201010000/814.013.pdf.

Bürgin, F. Rauscher & Stöcklin AG, E-Mail vom 12.2.2002, 2002.

BUWAL. Schutz vor Umweltschäden durch PCB-haltige Kondensatoren und Transformatoren. Schriftenreihe Umweltschutz Nr. 90. Technical report, Bundesamt für Umweltschutz, Bern, Switzerland, 1988.

BUWAL. Sonderabfälle in der Schweiz: Mengen und Zusammensetzung 1991. Schriftenreihe Umwelt Nr. 221. Technical report, Bundesamt für Umwelt, Wald und Landschaft, Bern, Switzerland, 1994a.

BUWAL. Diffuse Quellen von PCB in der Schweiz. Schriftenreihe Umwelt Nr. 229. Technical report, Bundesamt für Umwelt, Wald und Landschaft, Bern, Switzerland, 1994b.

BUWAL. Richtlinie für die Entsorgung von Strahlschutt – Nachtrag vom April 1995. Technical report, Bundessamt für Umwelt, Wald und Landschaft, Bern, Switzerland, 1995.

BUWAL. Praxishilfe: PCB-Emissionen beim Korrosionsschutz. Technical report, Bundesamt für Umwelt, Wald und Landschaft, Bern, Switzerland, 2000.

BUWAL. Ausgewählte polybromierte Flammschutzmittel - Stoffflussanalyse. Technical Report 338, Bundesamt für Umwelt, Wald und Landschaft, Bern, 2002.

BUWAL. Richtlinie PCB-haltige Fugendichtungsmassen – Beurteilung des Handlungsbedarfs und Empfehlungen für das Vorgehen bei Bauten. Technical report, Bundesamt für Umwelt, Wald und Landschaft, Bern, Switzerland, 2003.

BUWAL. Klärschlammentsorgung in der Schweiz – Mengen- und Kapazitätserhebung,. Umwelt-Materialien Nr. 181. Technical report, Bundesamt für Umwelt, Wald und Landschaft, Bern, 2004.

Davis, J., Geyer, R., Ley, J., He, J., Clift, R., Kwan, A., Sansom, M., and Jackson, T. Time-dependent material flow analysis of iron and steel in the UK – Part 2. Scrap generation and recycling. *Resour. Conserv. Recycl.*, 51:118–140, 2007.

EEA. Movements of waste across the EU's internal and external borders. Technical report, European Environmental Agency (EEA), Copenhagen, Denmark, 2012. URL http://www.eea.europa.eu/publications/movements-of-waste-EU-2012.

EEA. EMEP/EEA air pollutant emission inventory guidebook 2016. Technical guidance to prepare national emission inventories. Technical report, European Environmental Agency (EEA), Copenhagen, Denmark, 2016. URL http://www.eea.europa.eu/publications/emep-eea-guidebook-2016/part-b-sectoral-guidance-chapters/5-waste.

Engler, M. Bestandsaufnahme und Entsorgungskonzept für PCB-haltige Reststoffe und Abfälle, Vortrag am VDI-Seminar "Schadstoff PCB" vom 14./15. März 1995, 1995.

Eugster, M., Chappot, A.-C., and Kasser, U. Schlussbericht – PCB in Kleinkondensatoren aus Elektro- und Elektronikaltgeräten. Technische Kontrollstellen SENS und SWICO. Technical report, 2007.

Fahrni, H. P. Von der wilden Deponie zu den Verbrennungsrückständen. In *KVA Rückstände der Schweiz – Der Rohst. mit Mehrwert*. Bern, Switzerland, 2010.

Fahrni, H.-P. Sewage Sludge Disposal in Switzerland. Technical report, 2011. URL http://www.vivis.de/phocadownload/Download/2011_wm/2011_WM_673_682_Fahrni.pdf.

Harrad, S. J., Sewart, A. P., Alcock, R., Boumphrey, R., Burnett, V., Duarte-Davidson, R., Halsall, C., Sanders, G., Waterhouse, K., Wild, S. R., and Jones, K. C. Polychlorinated Biphenyls (PCBs) in the British Environment: Sinks, Sources and Temporal Trends. *Environ. Pollut.*, 85:131–146, 1994.

Hauser, A. Thesenpapier: Entsorgung beschichteter Stahlschrott. Bundesamt für Umwelt (BAFU). Holinger AG. Technical report, Bern/Luzern, Switzerland, 2014.

Hauser, A. Personal communication, June 2016, 2016.

Helton, J. C. and Davis, F. Latin hypercube sampling and the propagation of uncertainty in analyses of complex systems. *Reliab. Eng. Syst. Saf.*, 81(1):23–69, 2003. ISSN 09518320. doi: 10.1016/S0951-8320(03)00058-9.

Ifeu. Ermittlung von Emissionen und Minderungsmaßnahmen für persistente organische Schadstoffe in der Bundesrepublick Deutschland (UBA-FB 98-115). Technical report, Institut für Energie- und Umweltforschung Heidelberg, 1998. URL http://www.dioxindb.de/dokumente/UBA_1998_Texte_74-98_POPs_Inventare_PCB_SCCP_HCBD_gedruckt.pdf.

Jartun, M., Ottesen, R. T., Steinnes, E., and Volden, T. Painted surfaces - Important sources of polychlorinated biphenyls (PCBs) contamination to the urban and marine environment. *Environ. Pollut.*, 157(1):295–302, 2009. ISSN 1873-6424. doi: 10.1016/j.envpol.2008.06.036.

Kohler, M., Tremp, J., Zennegg, M., Seiler, C., Minder-Kohler, S., Beck, M., Lienemann, P., Wegmann, L., and Schmid, P. Joint sealants: An overlooked diffuse source of polychlorinated biphenyls in buildings. *Environ. Sci. Technol.*, 39(7):1967–73, 2005. ISSN 0013-936X. doi: 10.1021/es048632z.

KSEntsorgung. Daten und Fakten - Wissenswertes aus dem Unternehmen, 2017. URL http://www.ks-entsorgung.com/de/about/daten.html.

Kuhn, E. and Arnet, R. PCB in Vorschaltgeräten von Fluoreszenzlampen – eine Bilanzierung. Kantonales Labor Kanton Aargau. Technical report, Aarau, Switzerland, 1998.

Külling, D. R., Stadelmann, F. X., and Candinas, T. Nährstoffe und Schwermetalle im Klärschlamm 1975-1999. *Agrar Forsch.*, 9(5):200–205, 2002.

Kupper, T. Belastung und Quellen organischer Schadstoffe im Kärschlamm und ihre Bedeutung im Zusammenhang mit dem Ausstieg aus der landwirtschaftlichen Klärschlammverwertung in der Schweiz. *Osterr. Wasser- und Abfallwirtschaft*, 60(3-4):45–54, 2008. ISSN 0945358X. doi: 10.1007/s00506-008-0151-4.

Mackay, D. and Paterson, S. Evaluating the Multimedia Fate of Organic Chemicals: A Level III Fugacity Model. *Environ. Sci. Technol.*, 25(3):427–436, 1991.

MacLeod, M., Fraser, A. J., and Mackay, D. Evaluating and expressing the propagation of uncertainty in chemical fate and bioaccumulation models. *Environ. Toxicol. Chem.*, 21(4):700–9, 2002. ISSN 0730-7268. doi: 10.1002/etc.5620210403.

Marcomini, A., Capel, P. D., Lichtensteiger, T., Brunner, P. H., and Giger, W. Behavior of Aromatic Surfactants and PCBs in Sludge-Treated Soil and Landfills. *J. Environ. Qual.*, 18(4):523–528, 1989. ISSN 00472425. doi: 10.2134/jeq1989.00472425001800040024x.

Mengon, W. and Schlatter, C. Polychlorierte Biphenyle (PCBs) aus dauerelastischen Dichtungsstoffen in schweizerischer Innenraumluft. *Mitteilungen aus dem Gebiet der Leb. und Hyg.*, 84:250–262, 1993.

Morf, L., Buser, A. M., and Taverna, R. Dynamic Substance Flow Analysis Model for Selected Brominated Flame Retardants as a Base for Decision Making on Risk Reduction Measures (FABRO) – Final Report. GEO Partner AG Resource Management. Technical report, Zürich, Switzerland, 2007.

Persson, N. J., Pettersen, H., Ishaq, R., Axelman, J., Bandh, C., Broman, D., Zebühr, Y., and Hammar, T. Polychlorinated biphenyls in polysulfide sealants - Occurrence and emission from a landfill station. *Environ. Pollut.*, 138: 18–27, 2005.

Sakai, S., Hiraoka, M., Takeda, N., and Shiozaki, K. Coplanar PCBs and PCDDs/PCDFs in municipal waste incineration. *Chemosphere*, 27(1-3):233–240, 1993.

Sakai, S., Ukai, T., Takatsuki, H., Nakamura, K., Kinoshita, S., and Takasuga, T. Substance flow analysis of coplanar PCBs released from waste incineration processes. *J. Mater. Cycles Waste Manag.*, 1(1):62–74, 1999. doi: 10.1007/s10163-999-0008-3.

Schwarzenbach, R. P., Gschwend, P. M., and Imboden, D. M. *Environmental Organic Chemistry.* Wiley-Interscience, 2003.

SMDK. Portrait der Sondermülldeponie Kölliken (SMDK), 2016. URL www.smdk.ch.

Sundahl, M., Sikander, E., Ek-Olausson, B., Hjorthage, A., Rosell, L., and Tornevall, M. Determinations of PCB within a project to develop cleanup methods for PCB-containing elastic sealant used in outdoor joints between concrete blocks in buildings. *J. Environ. Monit.*, 1(4):383–387, 1999. ISSN 1464-0325. doi: 10.1039/A902528F.

Tremp, J. and Wegmann, L. Stand des 'Phase-out' von PCB in der Schweiz: Ergebnisse einer Untersuchung über PCB-Reservoire und Massnahmen für die beschleunigte und sichere Entsorgung. Arbeitsexemplar für die Schriftenreihe Umwelt. Technical report, Bern, Switzerland, 2001.

UNEP. Toolkit for Identification and Quantification of Releases of Dioxins, Furans and Other Unintentional POPs – under Article 5 of the Stockholm Convention. United Nations Environmental Programme (UNEP). Technical report, Nairobi, Kenia, 2013.

USEPA. Locating And Estimating Air Emissions From Sources Of Polychlorinated Biphenyls (PCB). U.S. Environmental Protection Agency (USEPA). Office Of Air And Radiation. EPA-450/4-84-007n. Technical report, North Carolina, USA, 1987.

Van den Berg, M., Birnbaum, L. S., Bosveld, A. T. C., Brunström, B., Cook, P., Feeley, M., Giesy, J. P., Hanberg, A., Hasegawa, R., Kennedy, S. W., Kubiak, T., Larsen, J. C., Van Leeuwen, F. X. R., Liem, A. K. D., Nolt, C., Peterson, R. E., Poellinger, L., Safe, S., Schrenk, D., Tillitt, D., Tysklind, M., Younes, M., Wærn, F., and Zacharewski, T. Toxic equivalency factors (TEFs) for PCBs, PCDDs, PCDFs for humans and wildlife. *Environ. Health Perspect.*, 106(12):775–792, 1998. ISSN 00916765. doi: 10.1289/ehp.98106775.

Van den Berg, M., Birnbaum, L. S., Denison, M., De Vito, M., Farland, W., Feeley, M., Fiedler, H., Hakansson, H., Hanberg, A., Haws, L., Rose, M., Safe, S., Schrenk, D., Tohyama, C., Tritscher, A., Tuomisto, J., Tysklind, M., Walker, N., and Peterson, R. E. The 2005 World Health Organization reevaluation of human and mammalian toxic equivalency factors for dioxins and dioxin-like compounds. *Toxicol. Sci.*, 93(2):223–241, 2006. ISSN 10966080. doi: 10.1093/toxsci/kfl055.

WebDab. WebDab search - Officially reported emission data, 2016. URL http://webdab.umweltbundesamt.at.

Wegmann, L. PCB in Blindstrom-Kompensationsanlagen - Bericht über die stichprobenweise Kontrolle von gewerblichen Kompensationsanlagen im Niederspannungsbereich im Zeitraum 2002-2004 - Entwurf. Kanton Basel-Landschaft. Technical report, Basel, Switzerland, 2005.

WorldBank. World Bank Open Data, 2016. URL http://data.worldbank.org.

Zennegg, M., Munoz, M., Schmid, P., and Gerecke, A. C. Temporal trends of persistent organic pollutants in digested sewage sludge (1993-2012). *Environ. Int.*, 60:202–208, 2013. ISSN 01604120. doi: 10.1016/j.envint.2013.08.020.
